# Exploration of tropocollagen unwinding by cathepsin K dimer with chondroitin 4-sulphate through microsecond timescale molecular dynamics

**DOI:** 10.1080/14756366.2026.2704785

**Published:** 2026-07-26

**Authors:** Mengyi Shan, Yue Wu, Chen Jiang, Jing Chen, Gang Cheng, Lu-Ping Qin

**Affiliations:** a School of Pharmaceutical Sciences, The First Affiliated Hospital of Zhejiang Chinese Medical University, Hangzhou, China; b QuanMin RenZheng (HangZhou) Technology Co., Ltd, Hangzhou, China; c College of Pharmaceutical Sciences, Zhejiang University, Hangzhou, Zhejiang, China; d Zhejiang Key Laboratory of Chinese Medicine Modernization, Zhejiang Chinese Medical University, Hangzhou, China

**Keywords:** Molecular dynamics, cathepsin K, tropocollagen, unwinding

## Abstract

Cathepsin K (CatK), a promising therapeutic target for bone-related diseases, uniquely unfolds triple-helical collagen and digests tropocollagen into soluble peptides in the presence of chondroitin 4-sulphate (C4-S). However, the molecular mechanism of CatK-mediated collagenolysis remains poorly understood, hindering the rational design of selective inhibitors. In this study, we performed microsecond-scale molecular dynamics simulations of a fully solvated ternary complex comprising the CatK dimer, tropocollagen segment, and C4-S, to indicate the structural and dynamical basis of tropocollagen unwinding. The process was initiated by the progressive disruption of six key inter-chain hydrogen bonds within the tropocollagen. C4-S adopted a cosine-like conformation that bridged CatK and tropocollagen, thereby stabilising the ternary complex. Unwinding occurred at the active site cleft, with Cys^25^ and Trp^184^ serving as critical residues that may contribute hydrogen bond disruption and substrate stabilisation. Our findings provided mechanistic insights into CatK-dependent collagen degradation and rational development of next-generation CatK inhibitors.

## GRAPHICAL ABSTRACT

**Figure:**
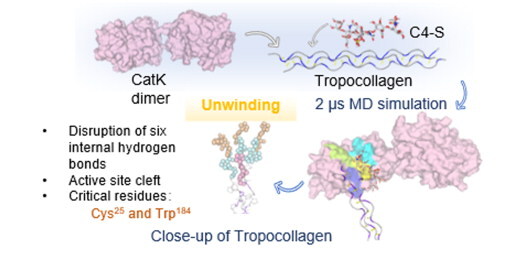

## Introduction

**Figure 1.**
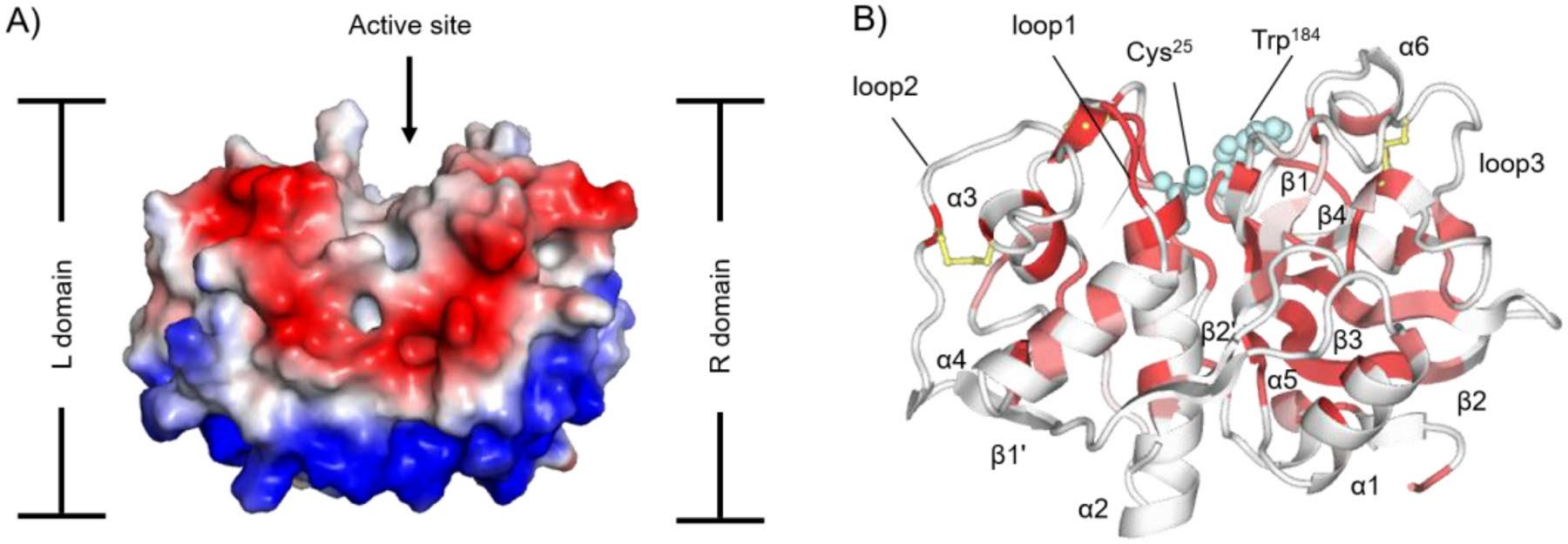
(A) Electrostatic surface potential analysis of CatK (PDB ID: 3C9E). Negative potential regions displayed in red, positive potential regions in blue. The electrostatic surface potential was generated with PyMOL. (B) The sequence homology of CatK mapped onto its three-dimensional structure. The colour ramping from white to red represents areas of weak and strong sequence conservation. Yellow sticks represent disulphide bonds between cysteine residues and cyan spheres indicate residues Cys^25^ and Trp^184^.

Cathepsin K (CatK) is a lysosomal cysteine protease belonging to the papain family, which includes 11 members (cathepsins B, C, F, H, K, L, O, S, V, W, and Z)^1^. These enzymes share a conserved papain-like fold and a catalytic triad composed of Cys-Asn-His residues. CatK is selectively expressed in osteoclasts, where it plays a pivotal role in bone resorption by degrading key components of the organic bone matrix, most notably triple helical collagen^2^. Synthesised as an inactive zymogen (procathepsin K), CatK undergoes proteolytic removal of its N-terminal propeptide under acidic conditions to yield the mature, enzymatically active form—a process that is autocatalytic and enhanced by chondroitin sulphate, a glycosaminoglycan (GAG) abundant in bone and cartilage^3–5^. The mature enzyme adopts a characteristic papain-like fold, composed of two domains designated the left (L) and right (R) domains (Figure 1(A)). Despite high sequence homology with other cysteine cathepsins^6^ (Figure 1(B)), CatK is uniquely capable of efficiently degrading triple-helical collagen during bone resorption^2^. This distinctive activity underpins its status as a promising therapeutic target for bone resorption disorders such as osteoporosis.

Collagen constitutes over 90% of the bone matrix’s organic component and is found in tendons, skin, blood vessels, and dentin^7^. Individual collagen triple helices are known as tropocollagen. Its triple-helical structure features repeating Gly-X-Y units, commonly Gly-Pro-Pro or Gly-Pro-Hyp (where hydroxyproline results from posttranslational modification of proline)^8^. CatK’s collagenolytic activity relies on the formation of complexes with GAGs^9^^,^^10^, such as chondroitin 4-sulphate (C4-S). In contrast to the physiological cleavage of covalent bond in collagen by collagenases of the matrix metalloproteinase (MMP) family^11^, CatK dimers primarily cause the disruption of hydrogen bonds among the three chains to unfold the triple helix^12^.

The tropocollagen unwinding mechanism, as described by Brömme *et al.*^13^^,^^14^, begins with C4-S adsorbing onto tropocollagen. The narrow active site entrance of CatK (∼5 Å) cannot accommodate the 15 Å-wide tropocollagen triple helix. However, positively charged residues electrostatically interact with C4-S, promoting the formation of a complex with tropocollagen hydrolysis activity^15^. Each hexasaccharide segment of C4-S binds with a CatK dimer^5^. CatK dimer binds to a tropocollagen–GAG complex which is unfolded by Gln^21^ and Gln^92^ before being broken by Cys^25^ ^12^.

Over the past two decades, despite approximately 5,000 patents filed for CatK inhibitors^16^, no such inhibitor has been approved for market. Notably, Balicatib^17^ and Odanacatib^18^, promising candidates for CatK inhibition, advanced to Phase II and Phase III clinical trials, respectively, but failed to gain regulatory approval. The role of CatK in collagen degradation remains not fully understood, with its biological significance and underlying mechanisms yet to be fully elucidated. Recent advances in computing power and *in silico* molecular interactions techniques allow for detailed investigation of the CatK’s role in the unwinding of tropocollagen. In this study, we employed microsecond timescale molecular dynamics (MD) simulations to model the unwinding of triple-helix tropocollagen. The simulations may indicate that tropocollagen unfolding involved the disruption of internal hydrogen bonds at the CatK active site, leading to structural unwinding. By integrating root mean square fluctuation analysis, saturation mutagenesis, and MD simulations, we suggested Cys^25^ and Trp^184^ as critical residues for these hydrogen bonds disruption.

## Materials and methods

### Three dimensional of protein structures

The three-dimensional protein structures of tropocollagen (PDB ID: 3AH9^19^ and 7CWK^20^) and CatK with C4-S (PDB ID: 3C9E)^21^ were obtained from the Protein Data Bank^22^ (PDB), https://www.rcsb.org. The triple-helical tropocollagen model peptide (PDB ID: 3AH9) represents a tropocollagen segment comprising approximately 28 amino acids in a repeating Gly-Pro-Pro sequence. The structure with PDB ID 7CWK consists of a specific sequence (Pro-Hyp-Gly)_3_-Gln-Arg-Gly-Glu-Arg-Gly-Phe-Pro-Gly-(Pro-Hyp-Gly)_3_. Firstly, the minimised energy conformation of the tropocollagen + C4-S dynamic structure was obtained through 1 µs MD simulation in aqueous solution. Following this, the CatK dimer + tropocollagen + C4-S complex was assembled by the 3D Builder module in Schrödinger suite 2022.

### Molecular dynamics simulations and analysis

The structures were prepared using the protein preparation wizard (Schrödinger suite 2022) with the standard settings, which added hydrogens, removed water and minimised the energy of the structure. The model is appropriate for pH 5.5 ± 2.0^23^, corresponding to the optimal conditions of CatK.

All MD simulations were performed using Desmond^24^ molecular dynamics software. Each system was solvated in an orthorhombic box with a minimum edge distance set to 10 Å. The box was filled with water molecules (TIP3P model), and Cl^-^ ions were added randomly to neutralise the system^25^. The MD simulation was performed using 2 femtoseconds for the integration of the equation of motion. The simulation system was equilibrated within the NPT ensemble framework with a temperature of 310 K by Nose-Hoover thermostats and 1 bar of pressure by Martina-Tobias-Klein barostat technique. All simulations were carried out by employing OPLS4 force field^26^. The trajectory was recorded at 1000-ps intervals. Each simulation was performed in duplicate with different random seeds to ensure reproducibility.

The root mean square deviation (RMSD) values (backbone atoms), the solvent accessible surface area (SASA) values (all atoms), and root mean square fluctuations (RMSF) were computed using the Simulation Interactions Diagram module to evaluate the stability of the complexes. The Simulation Event Analysis module was conducted for the measurement of atom distance. All the plots were performed in jupyter notebook to run the python script using the modules numpy, pandas, sklearn, and matplotlib.

### Free energy landscape

The free energy landscape (FEL)^27^ serves as a valuable analytical instrument, illuminating the shifts in energy associated with conformations throughout MD simulations. It is derived through conformational sampling methods that explore near-native state conformations. The calculation of the FEL can be approximated by:

$$\Delta G(X)=-k_{B}T\text{lnP}(X)$$

$k_{B}$ represents the Boltzmann constant; T is the absolute temperature. The reaction coordinate X represents two collective variables that characterise specific properties and conformational changes of the system. The probability density function, denoted as P(X), delineates the likelihood of the system’s various conformational states.

The RMSD and radius of gyration (Rg) were extracted from the MD simulation trajectories as collective variables^28^. Utilising the ‘sham’ command within the GROMACS^29^ suite, these variables were subjected to a two-dimensional projection analysis. The density of points on these coordinates correlated with conformational occurrence probability and inversely with the free energy level. The point distribution was interpreted as the probability density P(X), further elucidating the two- dimensional FEL based on the system’s joint probability distributions.

### Saturation mutagenesis

The residues scanning calculations module^30^ was used to mutate the standard amino acids of CatKB at the tropocollagen contact surface to alanine. The calculations were carried out with Prime Molecular mechanics generalised born surface area (MM/GBSA), which employed an implicit solvation model. The results analysis was conducted through the residues scanning results module, where a positive value indicates that the parent protein exhibits stronger binding than the mutant protein. The alteration in protein binding affinity was derived from the comparative binding energies, as delineated by the following equation:

R+L→R·LΔG (bind)

$$R^{\prime }+L\to R^{\prime }\cdot L\Delta G^{\prime }\;(\text{bind})$$

ΔΔG (bind)=ΔG′(bind)−ΔG (bind)

R denotes the parent receptor, L represents the ligand, $R^{\prime }$ is the mutated receptor and ΔΔG (bind) is the change in binding affinity. R+L and $R^{\prime }+L$ correspond the separated receptor and ligand, while R·L and $R^{\prime }\cdot L$ represent the receptor-ligand complexes.

## Results

### CatK dimer + tropocollagen + C4-S complex formation

Since no experimental structure of the tropocollagen + C4-S complex is available in the PDB, we extracted the C4-S structure from PDB ID: 3C9E^21^ and used a tropocollagen structure from PDB ID: 3AH9^19^. The tropocollagen was positioned roughly parallel to the vertical axis of the C4-S. A 1 μs MD simulation was performed for the tropocollagen + C4-S complex. The conformation at the global minimum of the FEL, constructed using RMSD and Rg as collective variables, was selected as the representative structure (Figure S1). This structure (tropocollagen_sulphate_FEL.pdb, from the file 1_tropocollagen_chondroitin) was provided in the file “trajectory” of the Supplemental material.

**Figure 2.**
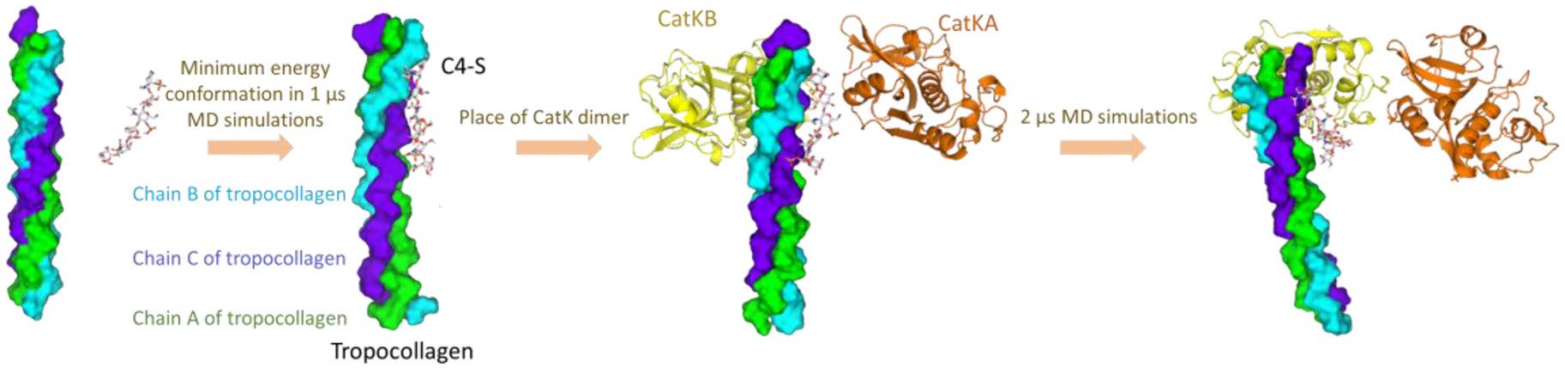
Workflow for the construction and MD simulation of the CatK dimer + tropocollagen + C4-S model. The two subunits of the CatK dimer, designated as CatKA and CatKB, are shown as orange and yellow cartoons, respectively. The three α-chains of tropocollagen (Chains A, B, and C) are depicted as green, blue, and purple molecular surfaces, while C4-S is shown as gray sticks.

In constructing CatK dimer + tropocollagen + C4-S (Figure 2), we referred to the previous model by Brömme *et al.*^12^. For dimer, we mirrored the CatK protein (PDB ID: 3C9E), then rotated it 180° clockwise along the vertical axis, and 180° upwards along the horizontal axis. The minimum energy conformation of tropocollagen + C4-S was placed into the groove of the dimeric CatK, the distance between the centroid of tropocollagen and CatK was measured at 31.3 Å. The tropocollagen was sandwiched between the C4-S and the CatKB’s residues Gln21 and Gln92 (Figure S2). In addition, the dimers did not make contact with each other’s scaffolding subunits. The CatK dimer + tropocollagen + C4-S complex (0 ns.pdb, from the file “2_CatKdimer_tropocollagen_chondroitin”) was available in the file “trajectory” within the Supplemental material. The constructed CatK dimer + tropocollagen + C4-S model was investigated for the unfolding process through a 2 μs MD simulation.

### Conformational variation

**Figure 3.**
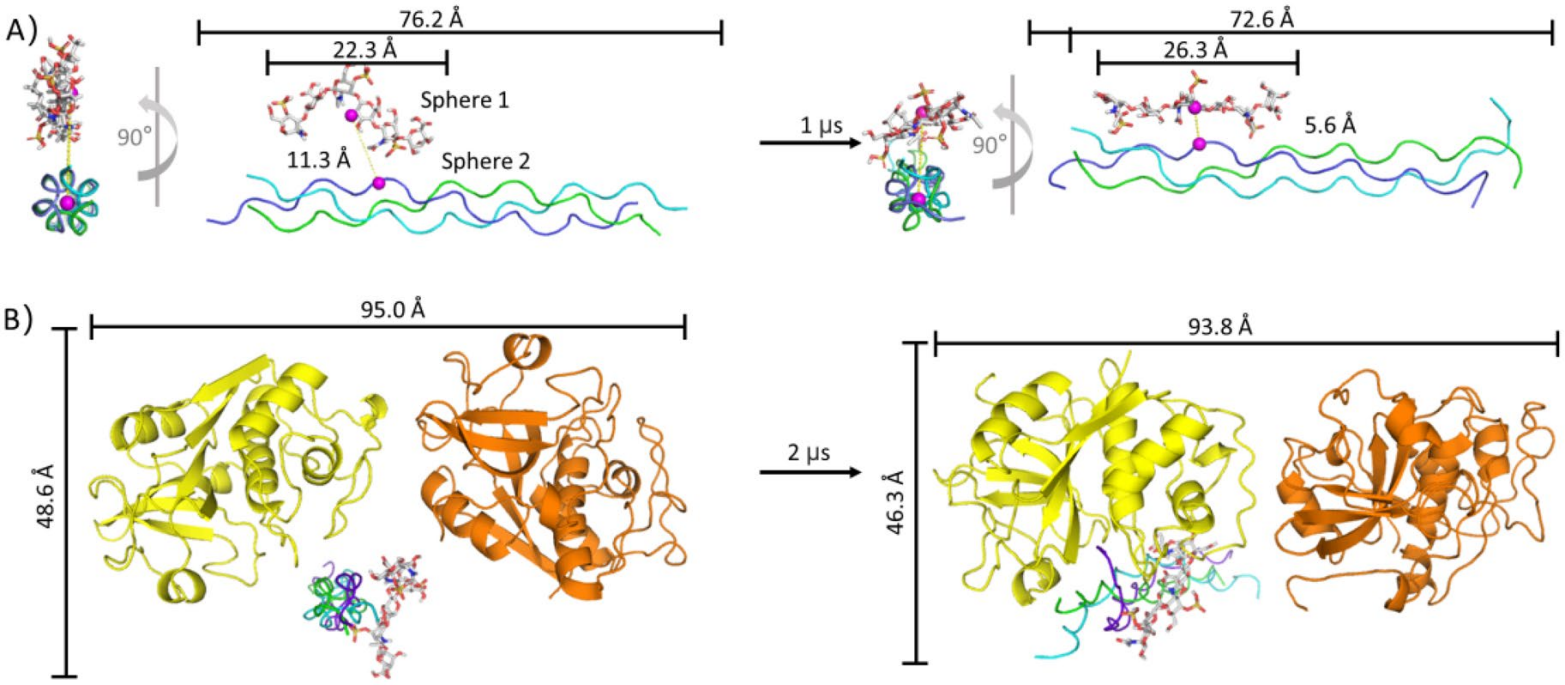
(A) The conformational change in tropocollagen + C4-S after 1 μs MD simulation. (B) The conformational change in CatK dimer + tropocollagen + C4-S after 2 μs MD simulation. CatKA and CatKB are shown in orange and yellow cartoons, respectively; tropocollagen Chains A, B, and C are depicted in green, blue, and purple cartoons; C4-S is shown as gray sticks. Pink sphere 1 represents the centre of mass of C4-S, and pink sphere 2 denotes the centre of mass of Pro^10^ on chain C.

To investigate transformation of the overall structure, we analysed the initial and final nanoseconds of the simulation. In tropocollagen + C4-S complex (Figure 3(A)), the centre of mass of C4-S was represented by pink sphere 1, while pink sphere 2 was placed at the centre of mass of Pro^10^, the closest residue on tropocollagen Chain C. The distance between these two spheres served as a measure of the proximity between C4-S and tropocollagen. Over the course of a 1 μs MD simulation, the complex underwent a significant conformational change, with the inter-sphere distance decreasing from 11.3 Å to 5.6 Å, indicating a progressive association between C4-S and the tropocollagen substrate. Water bridges played an important role in stabilising the interaction between tropocollagen and C4-S (Figure S3). Structural snapshots recorded at 200 ns intervals (Figure S4) further suggested the gradual tightening of the complex.

As shown in Figure 3(B), a pronounced reduction in the intermolecular distance between the two subunits of the CatK dimer (CatKA and CatKB) was observed during the 2 μs MD simulation of the CatK dimer + tropocollagen + C4-S system. Concurrently, the distance between tropocollagen and CatK decreased significantly, facilitating a favourable orientation for tropocollagen unwinding. The structural rearrangement suggested that CatK adopted a more compact and catalytically competent conformation, positioning itself optimally for substrate engagement and proteolytic activity.

### Trajectory analysis of CatK dimer + tropocollagen + C4-S complex

**Figure 4.**
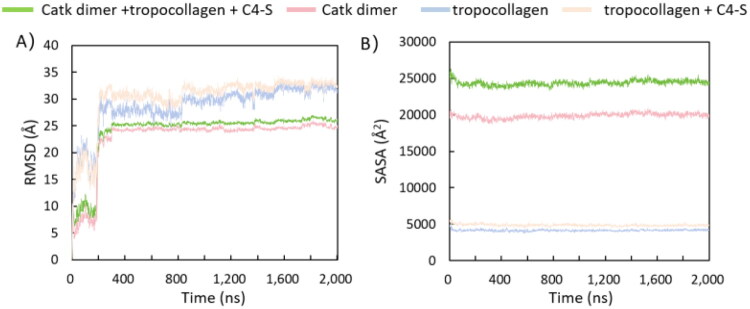
Temporal evolution of RMSD (A), and SASA (B) for CatK dimer + tropocollagen + C4-S ternary complex during MD simulations. The trajectory values for the CatK dimer + tropocollagen + C4-S complex are represented by a green line, while those for the CatK dimer, tropocollagen, and tropocollagen + C4-S components are shown as pink, blue, and light orange lines, respectively.

To evaluate the structural stability of the CatK dimer + tropocollagen + C4-S ternary complex, we calculated the RMSD of backbone atoms and the SASA over all atoms throughout the simulation trajectories in Figure 4 and Figure S5 (the replicate trajectory). The RMSD for the ternary complex was calculated relative to the initial conformation to monitor structural evolution (Figure 4(A)). An initial rise in RMSD observed within the 0–170 ns interval may be driven by the disruption of inter-chain hydrogen bonds in the tropocollagen, specifically between ChainB Gly^3^ and ChainC Pro^4^ as well as ChainC Gly^3^ and ChainA Pro^1^. This localised unwinding also altered the tropocollagen–CatK binding interface, triggering an increase in the RMSD of the CatK dimer (pink line). The notable increase in RMSD was observed around 185 ns, mainly attributed to the compaction of CatK into a C-shaped conformation. This conformational transition induced a cooperative compaction across the entire system, involving both the tropocollagen and the C4-S. After this event, the RMSD of CatK dimer + tropocollagen + C4-S ternary complex (green line) stabilised with 25.44 ± 0.94 Å. Furthermore, the tropocollagen (blue line) in the ternary complex showed an average RMSD of 28.58 ± 4.2 Å, higher than that in the single tropocollagen system (2.78 ± 0.55 Å, Figure S6) or tropocollagen + C4-S binary (3.60 ± 0.23 Å, Figure S7) system, reflecting the necessary structural movement required for tropocollagen to accommodate the binding of the CatK dimer.

SASA analysis provides insight into molecular exposure to the solvent. For the CatK dimer + tropocollagen + C4-S system (green line), the initial decrease in SASA suggested structural compaction in aqueous solution (Figure 4(B)). Subsequently, the SASA stabilised after 400 ns at an average value of 24384 ± 363 Å^2^. These trends collectively may indicate that the CatK dimer + tropocollagen + C4-S ternary complex underwent initial structural rearrangement and compaction, ultimately achieving a dynamically stable conformation that promoted localised unwinding of the tropocollagen triple helix.

### Structural snapshots of CatK dimer + tropocollagen + C4-S complex

**Figure 5.**
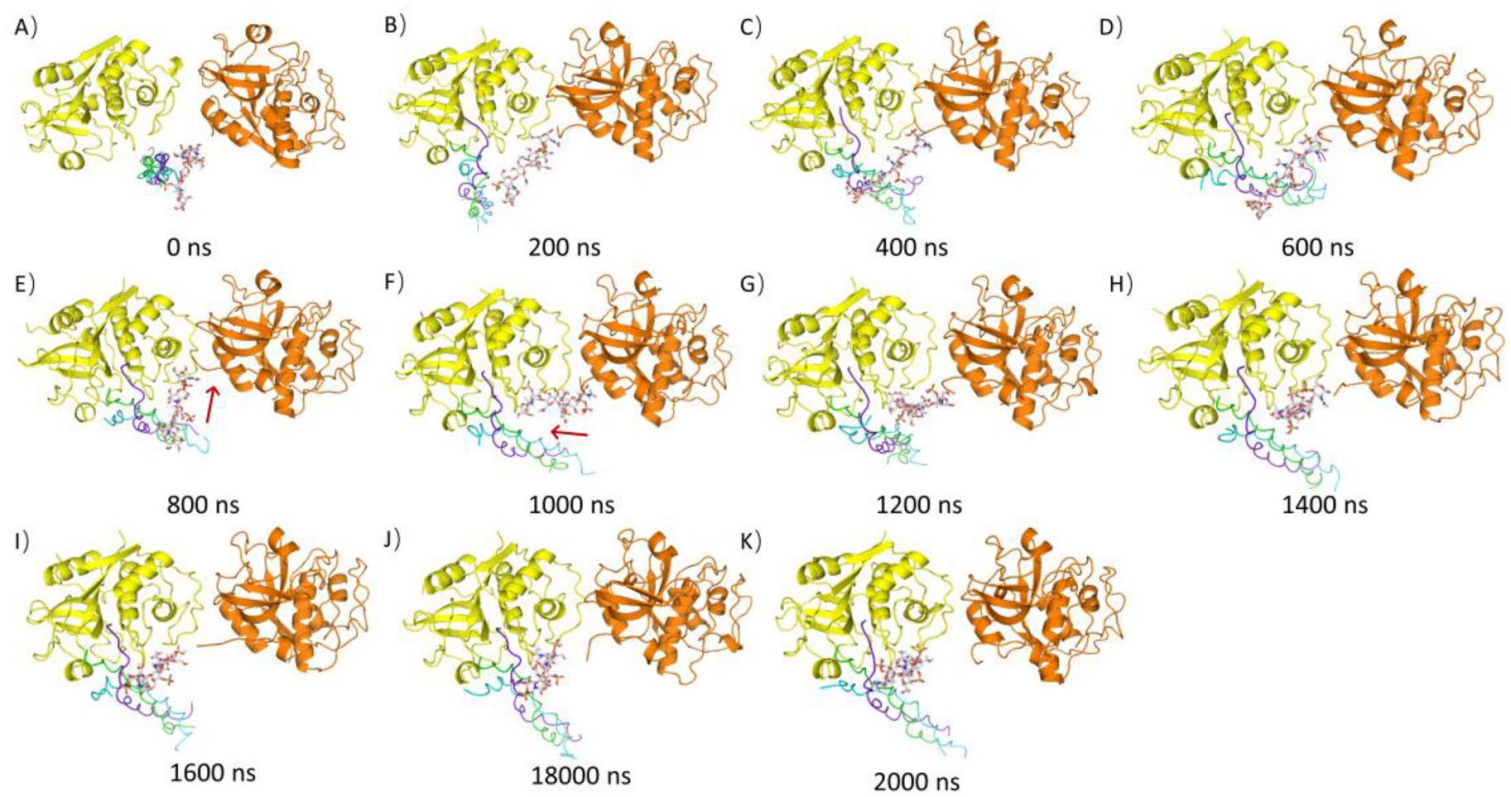
Time-resolved structural evolution of the CatK dimer + tropocollagen + C4-S complex during MD simulation (snapshots every 200 ns). The process captures tropocollagen Chain C insertion, C4-S conformational flip, and ternary complex stabilisation.

Structural snapshots were extracted at 200 ns intervals from the MD simulations to provide insights into the functional dynamics of CatK and the conformational rearrangements underlying its interaction with tropocollagen (Figure 5). An independent 2 μs MD simulation was also performed for validation, with corresponding snapshots presented in Figure S8. The full trajectories of two replicate simulations are provided in Movie S1 and Movie S2 of the Supplemental material, while Movie S3 illustrates a smoothed transition of tropocollagen unwinding from the initial to the final state.

The simulations displayed the rapid embedding of tropocollagen Chain C into the groove of CatKB, where it remained stably bound throughout the trajectory (Figure 5(B)). Unwinding initiated near the C-terminal nucleus of Chain C and propagated progressively along the peptide backbone, indicating that the triple helix dissociation occurred directly within the catalytic domain of CatK. Distance measurements may reflect that the presence of tropocollagen induced an expansion of the active site cleft, achieving an optimal separation of approximately 10 Å between tropocollagen and the CatK substrate binding site (Figure S9).

C4-S initially engaged the dimer interface through its first hexasaccharide unit, forming preliminary contacts with both CatK monomers. At around 1000 ns, a significant conformational flip occurred—characterised by a ∼180° rotation—where the lower face of C4-S became the upper face (Figure 5(F), arrow indicates rotational direction). By 1600 ns, C4-S adopted a fixed, bent conformation that bridged CatKB and tropocollagen (Figure 5(I)), thereby stabilising the ternary complex. This rotational transition likely represented a critical step in C4-S achieving its optimal binding mode. The initial interaction may reflect a sampling phase, during which C4-S to explore different orientations, followed by dynamic adaptation to the chemical and structural environment of the binding site—ultimately leading to a stable scaffold that facilitated complex assembly and collagenolysis.

During the early phase (first 200 ns), CatKA and CatKB moved closer, indicating dimer compaction. Compared to CatKA, CatKB exhibited more pronounced structural changes, including: 1) Loss of helices α1, α3, and α6; 2) Disruption of the β-sheet segment from Val^5^ to Asp^6^; 3) Conversion of loop3 into a β-sheet conformation. Conformational changes also affected residues at the termini of secondary structural elements, including Gly^67^ (α4), Glu^117^ and Ala^128^ (α5), and Ala^209^ (β2′). In CatKA, analogous but less extensive changes were observed: 1) Loss of helix α1; 2) Refolding of loop3 into a β-sheet; 3) Rearrangement of the β-sheet region (Arg^81^–Ile^83^). Local conformational changes were also observed in residues at the termini of helices and sheets, including Lys^42^ and Lys^43^ (α2), Gly^67^ and Tyr^79^ (α4), and Ala^126^ and Ala^128^ (α5).

Collectively, these observations suggest that the active site of CatK plays a central role in initiating tropocollagen triple helix unwinding, while C4-S acts as a molecular scaffold that stabilises the interaction between CatK and tropocollagen. By securing both CatK and tropocollagen, C4-S promotes the formation of complex, thereby enhancing tropocollagen unwinding process.

### Unwinding process of tropocollagen

The unwinding of tropocollagen was initiated by the disruption of inter-chain hydrogen bonds among the three polypeptide chains. As these interactions broke, the triple helix became structurally loosened, enabling increased chain flexibility, disordered motion, and enhanced accessibility for interactions with CatK. Self-interactions between exposed segments were also observed.

To distinguish transient hydrogen bond fluctuations from functionally persistent disruptions, we defined an “persistent disruption” as a continuous break lasting ≥100 ns, with no re-formation persisting for ≥100 ns thereafter. In comparison to a 2 μs MD simulation of a single tropocollagen molecule in explicit water, such prolonged disruptions of interchain hydrogen bonds were mostly absent (except for the hydrogen bond between ChainA Gly^2^ and ChainB Pro^1^ broken at 533 ns), as shown in Figure S10. It confirmed that the ≥100 ns threshold represented a meaningful criterion for persistent hydrogen bond disruption.

**Figure 6.**
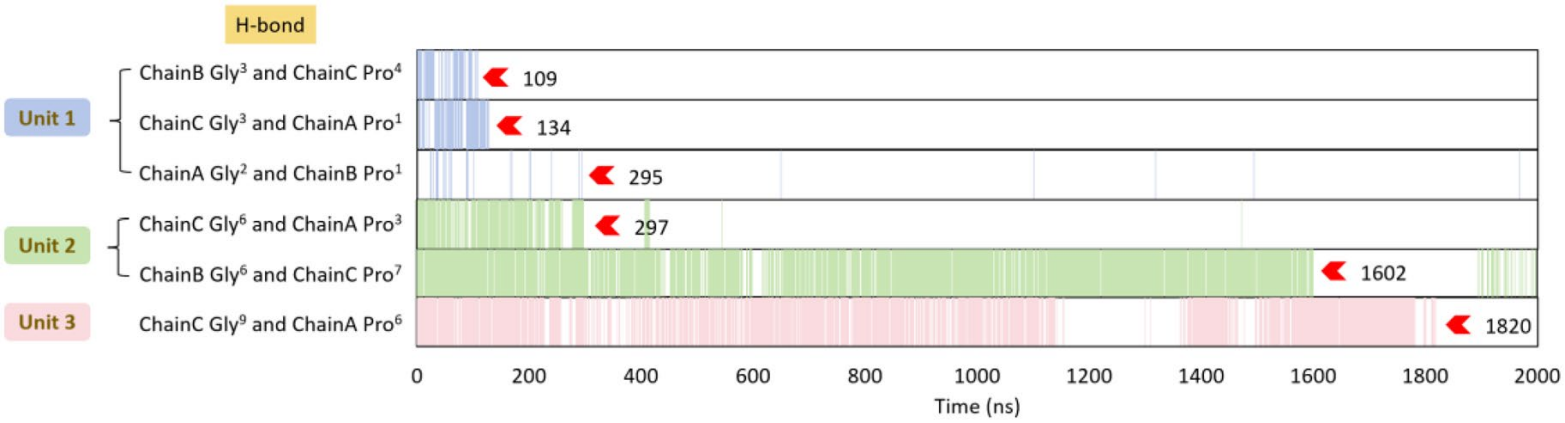
Temporal evolution and persistent disruption of interchain hydrogen bonds within the tropocollagen triple helix during the 2 μs MD simulation of the ternary complex (CatK dimer + tropocollagen + C4-S). Red arrows mark the onset of persistent disruption (defined as a break lasting ≥100 ns), with the specific time (ns) labelled.

**Figure 7.**
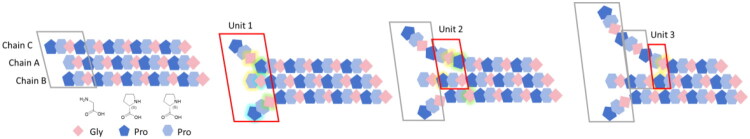
Schematic illustrations of the tropocollagen unfolding process. In the unit of Gly-Pro-Pro triplet, quadrilateral represents residue Gly, while the second and third residue Pro are represented by the pentagon and hexagon, respectively. The corresponding luminescent colours highlight the breaking of internal hydrogen bonds.

Over the 2 μs MD simulation, breakage of six hydrogen bonds was observed (Figure 6). The unfolding process from the repeating sequence of each chain was illustrated in Figure 7. Treating each Gly-Pro-Pro triplet as a single repeating unit, we observed that the first unit lost the internal hydrogen bonds within 300 ns. Regarding the Unit 2, the hydrogen bond between ChainC Gly^6^ and ChainA Pro^3^ underwent continuous breaking for 100 ns starting at 297 ns. Although it briefly re-formed thereafter, the restored state was transient (<100 ns), suggesting instability. The hydrogen bond between ChainB Gly^6^ and ChainC Pro^7^ exhibited rapid breaking and re-forming during the 0–1602 ns interval, indicative of a dynamic equilibrium. It only transitioned to a persistent state after 1602 ns, indicating localised unwinding. However, the hydrogen bond between ChainA Gly^5^ and ChainB Pro^4^ remained stable for 93.5% of the simulation time, with any break being transient (<100 ns). In Unit 3, only the ChainC Gly^9^ and ChainA Pro^6^ bond underwent long-lived rupture after 1820 ns.

These observations suggested that the formation and break of hydrogen bonds existed as dynamic equilibria. In tropocollagen unwinding process, breaks within the same unit tended to occur during similar time intervals, whereas subsequent units exhibited significantly slower disruption and a higher propensity for re-formation. Moreover, Chain C unfolded more readily compared to the other two chains, due to its location within the active groove, enabling favourable interactions with nearby residues.

### Conformation and functional role of C4-S structure

Efficient tropocollagen unwinding by CatK requires the formation of an oligomeric complex with C4-S. After CatK dimer + tropocollagen + C4-S system reached a stable state, the C4-S formed a cosine-like conformation with CatK and tropocollagen. Specifically, the GlcA-5 and GalNAc-6 residues of C4-S were positioned within Chain C, within the binding region Gly^3^-Gly^9^ (Figure S11). On the CatK surface, C4-S was positioned near a positively charged segment (Lys^17^-Gln^21^) and the negatively charged regions (Asp^61^-Gly^65^ and Pro^88^-Glu^93^).

CatK dimer + tropocollagen complex model without C4-S was constructed and subjected to a 1 μs MD simulation to assess the impact of C4-S absence on the structural and dynamic behaviour of the complex. At 81 ns, the tropocollagen tilted towards the CatKA subunit, accompanied by a significant rearrangement of the CatK dimer conformation (Figure S12). Subsequently, stable hydrogen bond interactions formed between Chain C and the residues Arg^111^ and Glu^112^ on CatKA. Within the tropocollagen, the inter-chain hydrogen bonds at ChainA Gly^2^ with ChainB Pro^1^ and ChainB Gly^3^ with ChainC Pro^4^ showed transient disruptions (over 100 ns), but remained intact for the rest of the simulation.

**Figure 8.**
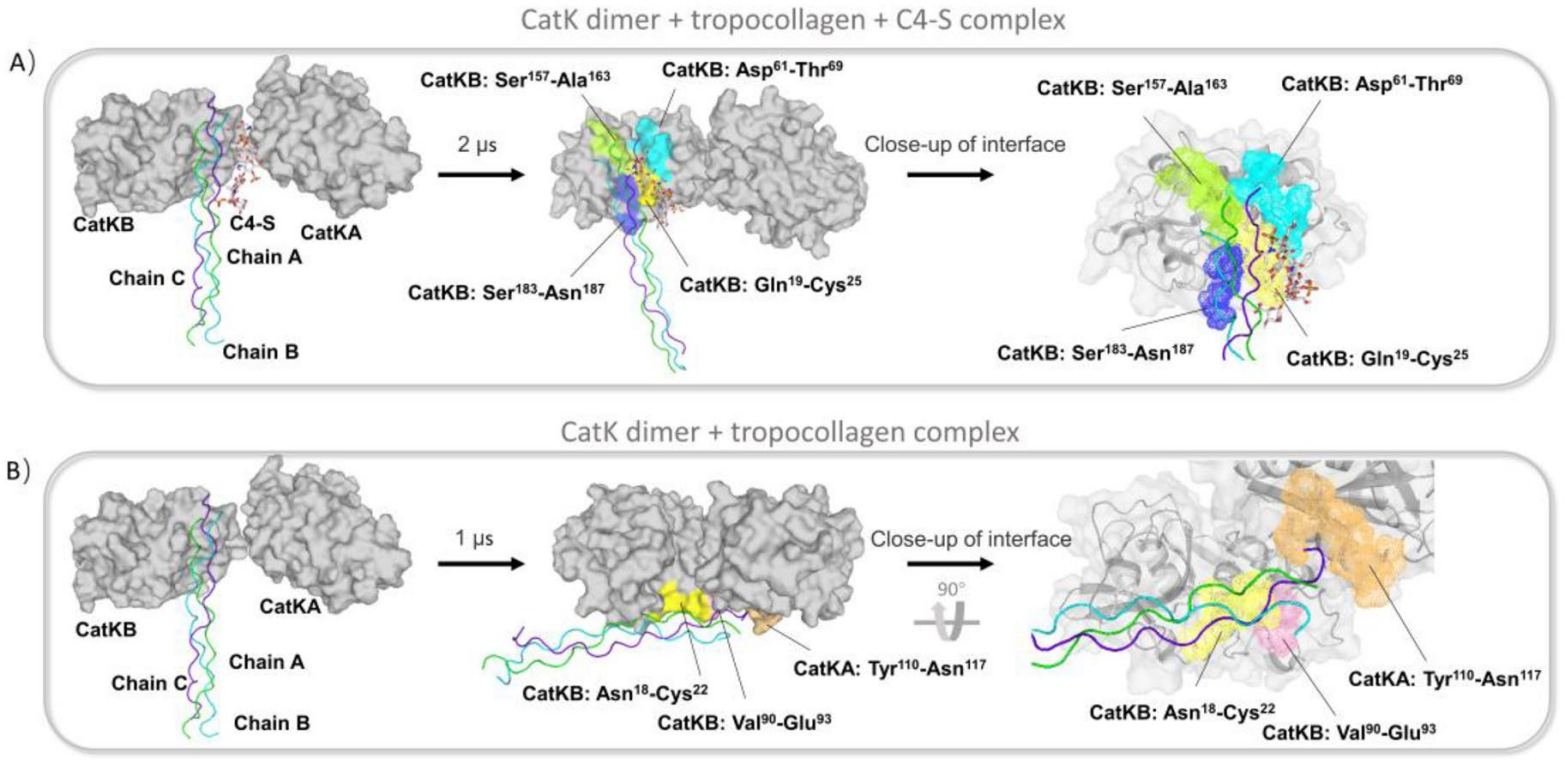
The interaction regions of Chain C as ligand with CatK dimer in CatK dimer + tropocollagen + C4-S complex (A) and CatK dimer + tropocollagen complex (B). CatKA and CatKB are displayed as gray surfaces; tropocollagen Chains A, B, and C are represented by green, blue, and purple cartoons, respectively; C4-S is illustrated as gray sticks. Interaction regions are highlighted with different coloured dots.

Furthermore, we compared the interaction interfaces between tropocollagen and the CatK dimer in two systems: CatK dimer + tropocollagen + C4-S and CatK dimer + tropocollagen. Chain C was selected as the ligand for the interaction analysis, owing to its unwinding degree and positioning within the CatK active site. Obviously, the absence of C4-S affected the Asp^61^-Thr^69^, Ser^157^-Ala^163^ and Ser^183^-Asn^187^ regions on CatKB (Figure 8), with loss of water bridges and hydrogen bonds to tropocollagen (Figure S13). Analysis of the RMSF showed that CatK dimer maintained RMSF values below 4.5 Å in the presence of C4-S (Figure S14), whereas these values increased to below 8.0 Å in C4-S absence (Figure S13), signifying CatK dimer instability without C4-S. Consequently, CatKB’s restraint on tropocollagen weakened, impairing the control over tropocollagen positioning for unwinding. These observations underscored the C4-S could contribute to stabilising the ternary complex and promoting unwinding efficiency.

### Key functional residues of CatK

Among the cathepsin family, CatK and CatL are the only two enzymes capable of breaking tropocollagen, with CatL exhibiting only a quarter of the potency of CatK^31^. The active site of CatK is composed of four subsites (S1’, S1, S2, and S3)^6^. According to previous studies^16^, the key amino acids (Gln^19^, Gly^23^, Cys^25^, Asp^61^, Tyr^67^, His^162^, Trp^184^, and Leu^209^) within the catalytic region of CatK, are commonly utilised in the design of new ligands. These eight residues were selected for detailed stability and binding analysis.

RMSF analysis revealed that Cys^25^ exhibited the highest stability (1.14 Å), consistent with earlier biochemical studies by Brömme *et al*.^12^. The other residues showed relatively higher flexibility: Gln^19^ (1.47 Å), Gly^23^ (1.66 Å), Asp^61^ (1.98 Å), Tyr^67^ (1.56 Å), His^162^ (1.37 Å), Trp^184^ (1.43 Å), and Leu^209^ (1.71 Å), more detail was shown in Figure S14. To further probe their functional importance, alanine-scanning mutagenesis was performed on these catalytic residues (Table S1). Binding affinity changes (ΔΔ*G*) were calculated using MM/GBSA. Among the eight residues, Trp^184^ exhibited the highest ΔΔ*G* value (12.22 kcal/mol), indicating its critical roles in substrate binding.

To explore the roles of Cys^25^ and Trp^184^ in collagenolytic activity, site-directed mutagenesis was performed to substitute each residue with alanine, yielding the C25A and W184A variants. After structural reconstruction and solvation, each system was subjected to a 1 μs MD simulation to assess the effects of these mutations on substrate binding and structural dynamics. The structural trajectory snapshots of the mutant systems (CatKA + mutant CatKB + tropocollagen + C4-S) were obtained from the trajectory file in the Supplemental material.

**Figure 9.**
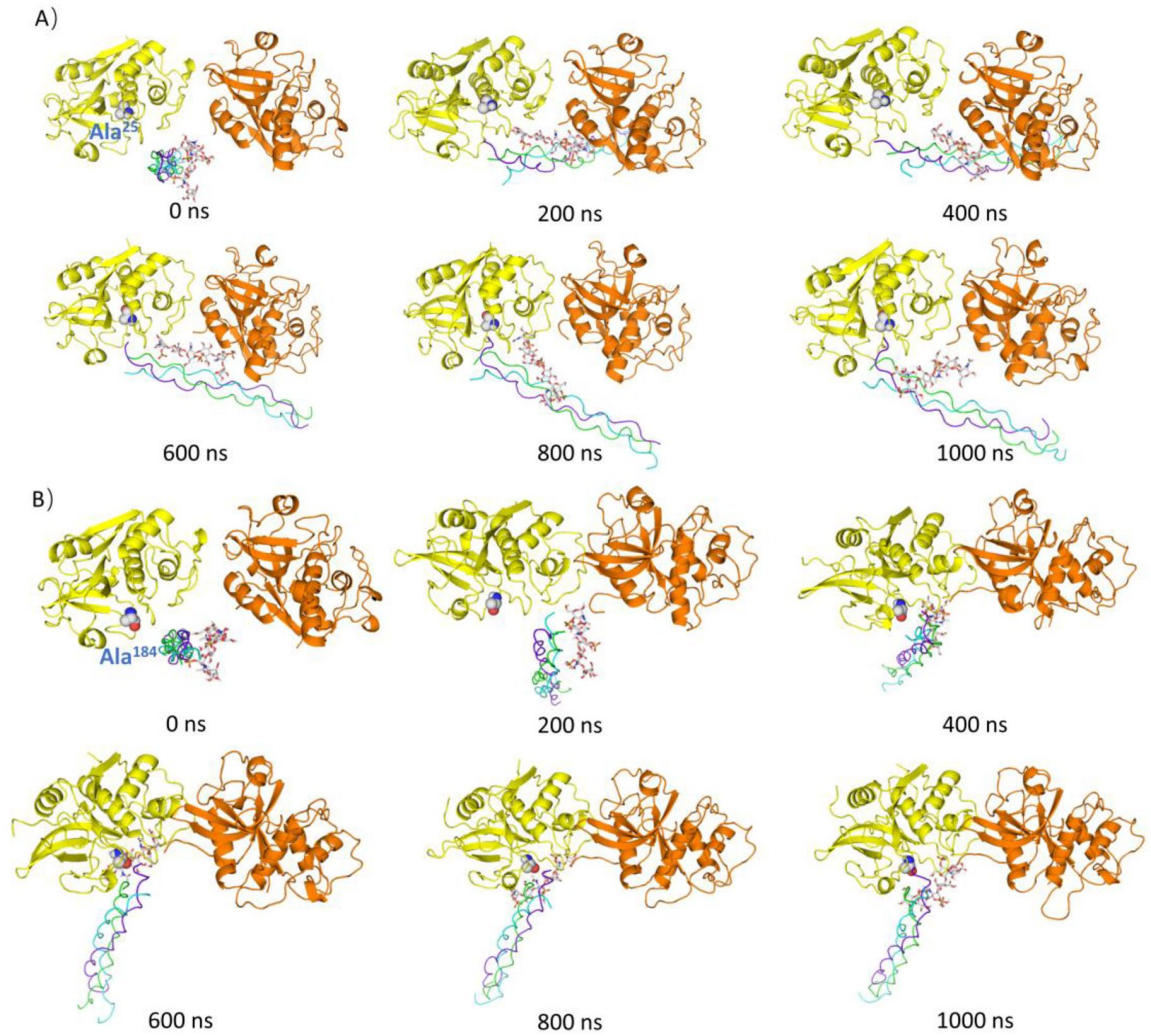
Structural snapshots of CatKA + mutant CatKB + tropocollagen + C4-S system at 200 ns intervals. (A) C25A mutant showing partial unwinding localised to Unit 1. (B) W184A mutant retaining triple-helical integrity with minimal structural perturbation. CatKA and CatKB are shown in orange and yellow, respectively; the residues Ala^25^ (A) and Ala^184^ (B) are shown as spheres.

For C25A mutant, tropocollagen was still able to enter the active site cleft, indicating that substrate recognition and initial binding were preserved (Figure 9(A)). Specifically, an interaction between Ala^25^ of CatK and Pro^1^ of ChainC was observed. However, the triple-helix unwinding process was severely limited. Throughout the 1 μs MD simulation, the only sustained dissociation occurred in the ChainC Gly^3^ and ChainA Pro^1^ contact at a late stage (∼591 ns), with no further unwinding observed (Figure S15).

For W184A mutant, the tropocollagen triple helix retained its overall integrity throughout the simulation, exhibiting only minor displacements (Figure 9(B)). Unwinding was largely restricted to Unit 1, with primary interaction breaks occurring between 250 and 300 ns (Figure S15). The rupture of the three hydrogen bonds in Unit 1 was delayed by approximately 100 ns compared to the wild-type system. These findings may indicate that Trp^184^ was not directly catalytic but played a key structural role in stabilising the open, unwound tropocollagen conformation via strong van der Waals interactions, thereby facilitating processive unwinding.

Overall, the simulations suggest that Cys^25^ is essential for initiating triple-helix destabilisation, while Trp^184^ is critical for maintaining the unwound state necessary for efficient unwinding.

### Conformation and function of CatK dimer interface

The CatK dimer is the functional unit in collagen degradation. Unlike mirror-symmetric dimer structures, the CatK dimer was formed by rotating one CatK molecule (CatKB) 180° around the vertical axis and 180° around the horizontal axis relative to the other (CatKA). We investigated the stability of the CatK dimer interface using the final nanosecond of the MD simulation. Consistent with Brömme *et al*.^12^, the CatK dimer adopted an elongated C-shaped conformation.

The negatively charged right domain of CatKB interacted strongly with the positively charged left domain of CatKA, forming a stable interface. This interaction was mediated primarily by hydrogen bonds and salt bridges involving residues Pro^100^, Asn^99^, Cys^96^, Ser^95^, and Glu^94^ from CatKB, and Asn^201^, Glu^118^, Asn^199^, Lys^176^, Lys^122^, and Arg^123^ from CatKA, as illustrated in Figure S11. Contact distances for these interactions are detailed in Table S2.

To further explore the role of the CatK dimer interface throughout the unwinding process, we performed a 1 μs MD simulation with CatKA removed. As shown in Figure S16, C4-S failed to stably bind to the CatKB surface, impairing tropocollagen anchoring. After 609 ns, tropocollagen dissociated from the active site cavity. Notably, the tropocollagen triple helix maintained the overall structural integrity throughout the simulation, with no persistent disruption of internal hydrogen bonds. These findings suggest that the CatK dimer interface is critical for constraining C4-S mobility, thereby facilitating tropocollagen association with CatKB.

### Influence of tropocollagen sequence variation on complex interfacial interactions

Apart from the Gly-Pro-Pro repeating motif, Gly-Pro-Hyp is a prevalent triplet in tropocollagen, accounting for approximately 10.5% of the total sequence^8^. Therefore, we performed additional 2 μs MD simulations for CatK dimer + tropocollagen_Gly-Pro-Hyp_ + C4-S system to explore the influence of alternative tropocollagen sequences (Figure S17). During the unwinding process, breakage of four hydrogen bonds was observed (Figure S18). Notably, the hydrogen bonds within Unit 1 broke within the first 100 ns. However, in Unit 2, only the bond between ChainA Pro^4^ and ChainB Gly^6^ disrupted at 989 ns. It suggested that the Gly-Pro-Hyp motif imparted greater structural stability to tropocollagen compared to the Gly-Pro-Pro counterpart, aligning with previous report indicating that a high Hyp/Pro ratio generally correlated with increased collagen stability^32^.

**Figure 10.**
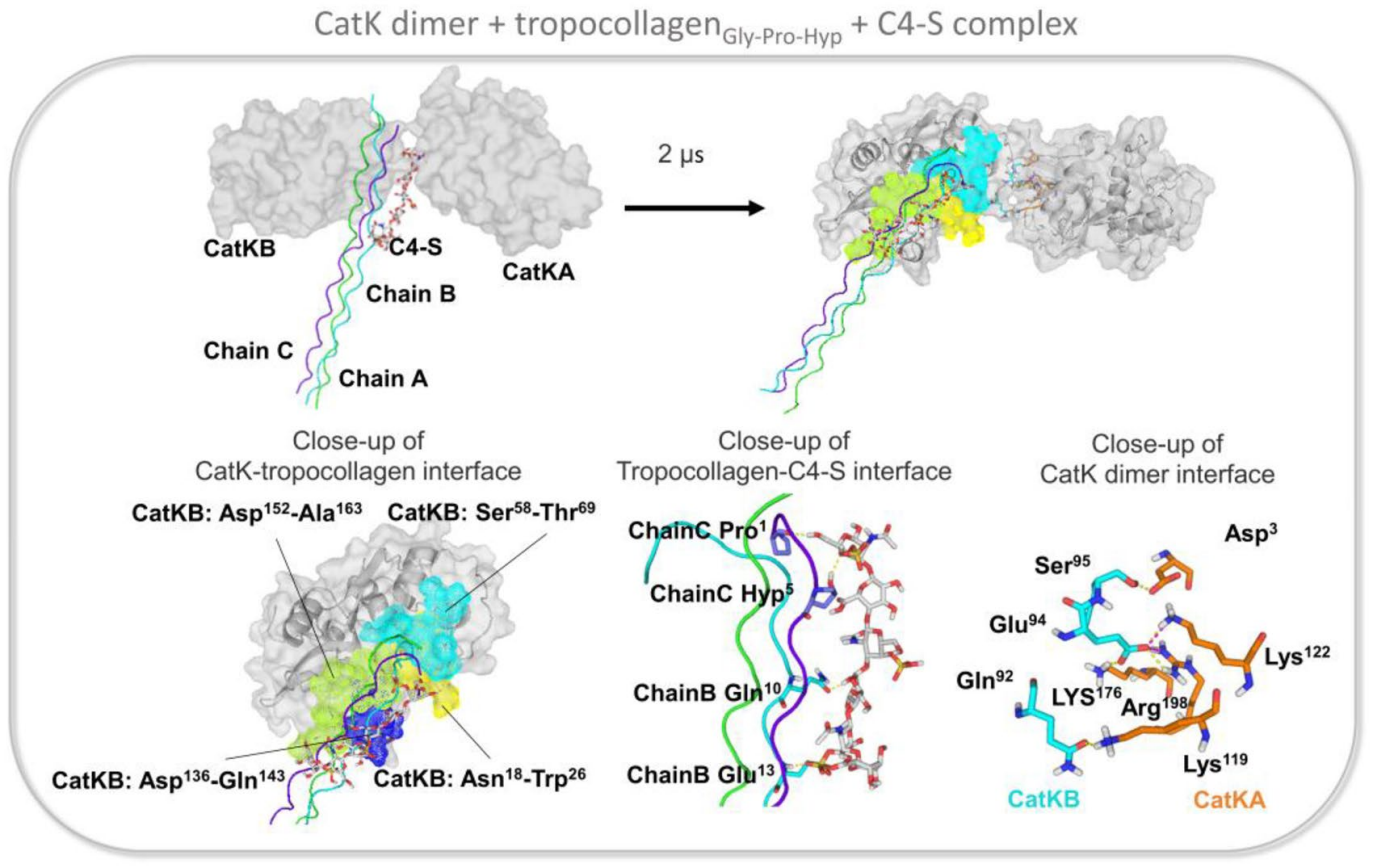
The interactions within the complex of CatK dimer, tropocollagen_Gly-Pro-Hyp_, and C4-S. CatKA and CatKB are displayed as gray surfaces; tropocollagen Chains A, B, and C are represented by green, blue, and purple cartoons, respectively; and C4-S is shown as gray sticks. For the CatK dimer–tropocollagen interface analysis, Chain B serves as the ligand, with specific interaction regions highlighted by multicoloured dots. For the CatK dimer interface analysis, the contacts between CatKA (right) and CatKB (left). Blue sticks represent CatKB interacting residues, orange sticks represent CatKA interacting residues, yellow dashed lines indicate hydrogen bonds, and purple dashed lines indicate salt bridges.

As illustrated in Figure 10, the unwinding site of the tropocollagen_Gly-Pro-Hyp_ remained positioned within the active site of CatK. Owing to its location within the active groove, Chain B unfolded more readily than the other two chains and was therefore selected as the ligand for the subsequent interaction analysis. While the tropocollagen interactions with regions (Asn^18^-Trp^26^, Ser^58^-Thr^69^, and Asp^152^-Ala^163^) of CatK were maintained, the binding interface shifted away from the Ser^183^-Asn^187^ region towards Asp^136^-Gln^143^. This transition may be driven by the hydrogen bond between Hyp and polar residues (like Thr^139^, Ser^140^, and Gln^141^), thereby altering the interface of the complex.

Variations in the tropocollagen sequence obviously affected the C4-S interaction interface, with loss of interface with CatK. The interaction region included ChainC Pro^1^-Arg^11^ and ChainB Pro^7^-Glu^13^ mediated predominantly by hydrogen bond: GalNAc-2 interacted with ChainB Glu^13^ GlcA-3 bond to ChainB Gln^10^ and GalNAc-6 engaged with ChainC Pro^1^ and ChainC Hyp^5^. It may indicate that the introduction of the Hyp strengthen the local interaction between tropocollagen and C4-S.

The CatK dimer interface remained virtually unaffected by the sequence variations in tropocollagen. This dimeric interface continued to be mediated by a network of hydrogen bonds and salt bridges involving residues Ser^95^, Glu^94^ and Gln^92^ from CatKB, and Arg^198^, Lys^176^, Lys^122^, Lys^119^ and Asp^3^ from CatKA (Table S3). Collectively, the Gly-Pro-Hyp motif of tropocollagen affected the unwinding rate and strengthened local interactions with C4-S, without altering the unwinding site or CatK dimeric assembly.

## Discussion

Previous computational and mechanistic studies related to hydrogen-bond disruption and structural destabilisation, focusing on factors such as water molecules^33^, temperature and pressure^34^, lipidation residues^35^, hydroxyproline content^36^ and terminal repeats^37^. However, these efforts were largely confined to the thermal or mechanical destabilisation of isolated tropocollagen segments. Consequently, the structural dynamics of binary or ternary CatK complexes, especially during enzymatic degradation, remain poorly characterised.

In contrast to MMP family collagenases, which cleaved the triple helix through hydrolysis of covalent peptide bonds^11^, CatK degraded tropocollagen by disrupting the interchain hydrogen bonds, leading to unwinding of the triple helix. Notably, efficient degradation by CatK required dimerisation and was facilitated by C4-S, which promoted the formation of proteolytically active oligomers^13^^,^^14^.

While current experimentally validated principles for CatK inhibitors primarily focused on targeting the active-site subsites^16^, candidates like Odanacatib^18^ and Balicatib^17^ were terminated due to severe side effects (e.g., cardiovascular events and skin toxicities). These adverse effects associated with active-site-directed CatK inhibitors likely stem from complete enzymatic blockade or off-target inhibition, which impaired the hydrolysis of non-extracellular matrix substrates in non-skeletal tissues^38^^,^^39^. Unlike active-site inhibitors, exosite inhibitors^39^^,^^40^ by Brömme *et al.* can selectively inhibit collagenase activity without affecting the enzyme’s active site by targeting the protein-protein^14^^,^^41^ and protein-GAG^5^ interfaces. Nevertheless, the atomic-level mechanism underlying this process remains elusive, limiting the rational design of selective inhibitors.

Our study modelled the structural dynamics of the CatK dimer + tropocollagen + C4-S ternary complex through microsecond timescale MD simulations. It suggested that CatK spontaneously trapped and initiated the unwinding of a tropocollagen strand, a process critically scaffolded by C4-S and the CatK dimer interface. The structural insights into these protein-protein and protein-GAG interfaces provide foundation for exosite mapping, thereby guiding the rational development of CatK inhibitors against bone resorption disorders. The MD simulations effectively displayed the physical dynamics of hydrogen bond disruption during unwinding, as it involved changes in interatomic distances. However, the activity of CatK relies on an acidic environment, indicating that the all components may undergo protonation state changes, including complex electronic rearrangements. Classical MD simulations, relying primarily on force fields, struggle to protonation processes, which involve alterations in chemical bonds. Therefore, a comprehensive understanding of the CatK unwinding mechanism, particularly concerning protonation and related chemical reactions, requires future studies to employ quantum Mechanics/Molecular Mechanics (QM/MM)^42^ or reactive force field molecular dynamics (ReaxFF MD)^43^ methods.

In the native bone matrix environment, tropocollagen forms microfibrils which exhibits limited mobility. The only plausible assembly site for a CatK dimer is at the gap region edges of the fibril^12^. In contrast, our current model lacked these structural constraints, allowing the molecules to move freely during the simulation. Consequently, when a hydrogen bond break, the absence of substrate fixation prevented CatK from effectively translocating to the next binding site. This likely hindered the persistent disruption of hydrogen bonds and allowed for rapid re-bonding. Therefore, future experiments should incorporate a constraint strategy for the tropocollagen to better model physiological conditions. Alternatively, applying an external force to the CatK dimer oriented it along the tropocollagen axis, simulating its potential directional movement. It remains possible that multiple CatK dimers can bind simultaneously and act along the collagen fibril, potentially influencing local unwinding. Additionally, since the CatK dimer constitutes the majority of the entire complex, minor changes in tropocollagen and C4-S during analysis might not be fully captured. To validate the proposed mechanism, site-directed mutagenesis^44^ could be employed to verify key residues, and hydrogen/deuterium exchange mass spectrometry^45^ could be utilised to monitor the dynamic disruption of inter-chain hydrogen bonds. Consequently, there remains wide scope for further exploration into the unwinding process.

## Conclusions

Our microsecond timescale MD simulations of the CatK dimer + tropocollagen + C4-S complex revealed that tropocollagen unwinding was initiated by the progressive disruption of six key inter-chain hydrogen bonds within the triple helix. This local unwinding was coupled with a conformational tightening of the CatK dimer, tropocollagen, and C4-S, which promoted the formation of new hydrogen bonds that strengthened enzyme–substrate interactions. Notably, C4-S adopted a bridging cosine-like conformation that facilitated complex assembly. Cys^25^ and Trp^184^ could contribute as critical residues for hydrogen bond disruption and substrate stabilisation, respectively. This work provided a detailed molecular level mechanism of CatK-mediated collagenolysis and delivered high-resolution structural models that can serve as starting points for exosite mapping and the rational design of next-generation CatK inhibitors for bone resorption disorders.

## Supplementary Material

Supplemental_material_2026_6_18.docx

## Data Availability

The authors confirm that the data supporting the findings of this study are available within the article and its supplementary materials. The structural trajectory snapshots and simulation movies in the study are openly available in the GitHub repository at https://github.com/AI-amateur/MD_CatK_C4-S_tropocollagen.
