# Supplementary material for: Exploration of tropocollagen unwinding by cathepsin K dimer with chondroitin 4-sulphate through microsecond timescale molecular dynamics
Source: J Enzyme Inhib Med Chem. 2026 Jul 26;41(1):2704785. doi: 10.1080/14756366.2026.2704785 (PMC13410547; doi:10.1080/14756366.2026.2704785)
Supplement: Supplemental_material_2026_6_18.docx [file IENZ_A_2704785_SM6846.docx]

Supplemental material

Exploration of Tropocollagen Unwinding by Cathepsin K Dimer with Chondroitin 4-sulfate through Microsecond Timescale Molecular Dynamics

**Table of Contents**

| **Sr.No.** | **Contents and characterization data of compounds** | **Page no.** |
| --- | --- | --- |
|  | **Figure S1** (A) Free energy landscape of tropocollagen with C4-S complex. (B) Representative minimum energy conformation of tropocollagen + C4-S complex. | 3 |
|  | **Figure S2** Structural construction for CatK dimer + tropocollagen + C4-S model. | 4 |
|  | **Figure S3** Molecular dynamics analysis of tropocollagen interactions with the C4-S. | 4 |
|  | **Figure S4** Time-resolved structural evolution of the tropocollagen + C4-S complex during MD simulation (snapshots every 200 ns). | 5 |
|  | **Figure S5** Temporal evolution of RMSD and SASA for CatK dimer + tropocollagen + C4-S ternary complex during a replicate molecular dynamics simulation. | 5 |
|  | **Figure S6** Temporal evolution of RMSD and SASA for tropocollagen system during MD simulations. | 6 |
|  | **Figure S7** Temporal evolution of RMSD and SASA for tropocollagen + C4-S binary complex during MD simulations. | 6 |
|  | **Figure S8** Time-resolved structural evolution of the CatK dimer + tropocollagen + C4-S complex during a replicate MD simulation (snapshots every 200 ns). | 7 |
|  | **Figure S9** Tropocollagen binding site geometry and dynamics in CatK. | 7 |
|  | **Figure S10** Temporal evolution and persistent disruption of interchain hydrogen bonds within the tropocollagen triple helix during the 2 μs MD simulation of the tropocollagen system. | 10 |
|  | **Figure S11** (A) Electrostatic surface potential of the CatK dimer. (B) 2D interaction diagram of C4-S within CatK dimer + tropocollagen + C4-S. (C) The contacts between CatKA and CatKB. | 10 |
|  | **Figure S12** Time-resolved structural evolution of the CatK dimer + tropocollagen complex during MD simulation (snapshots every 200 ns). | 11 |
|  | **Figure S13** Molecular dynamics analysis of tropocollagen Chains A, B and CatK dimer interactions with Chain C in CatK dimer + tropocollagen model. | 11 |
|  | **Figure S14** Molecular dynamics analysis of tropocollagen Chains A, B and CatK dimer interactions with Chain C in CatK dimer + tropocollagen + C4-S model. | 12 |
|  | **Table S1** The ΔΔ*G* values for alanine mutants of key CatK catalytic residues. | 13 |
|  | **Figure S15** Temporal evolution and persistent disruption of interchain hydrogen bonds within the tropocollagen triple helix during the 2 μs MD simulation of the CatKA + mutant CatKB + tropocollagen + C4-S system. | 14 |
|  | **Table S2** The protein-protein contacts between CatKA and CatKB within CatK dimer + tropocollagen + C4-S system. | 14 |
|  | **Figure S16** Time-resolved structural evolution of the CatKB + tropocollagen + C4-S complex during MD simulation (snapshots every 200 ns). | 15 |
|  | **Figure 17** Structural snapshots of CatK dimer + tropocollagen_Gly-Pro-Hyp_ + C4-S system at 200 ns intervals. | 15 |
|  | **Figure S18** Temporal evolution and persistent disruption of interchain hydrogen bonds within the tropocollagen triple helix during the 2 μs MD simulation of the CatK dimer + tropocollagen_Gly-Pro-Hyp_ + C4-S system. | 15 |
|  | **Table S3** The protein-protein contacts between CatKA and CatKB within CatK dimer + tropocollagen_Gly-Pro-Hyp_ + C4-S system. | 16 |

To further investigate the conformational interaction, the free energy landscape profile^1^ of the complex were studied. As illustrated in Figure S1A, the root mean square deviation (RMSD) with the radius of gyration (Rg) for tropocollagen + C4-S model showed the large regions of low energy, along with a global minimum energy region. In the conformation of minimum energy, C4-S primarily adsorbed along Chain C. The three hexasaccharide (GlcA-1, GalNAc-2 and GlcA-3) segment were nearly parallel to Chain C, with dihedral angles of 164.5° and 3.7° respectively. C4-S established hydrogen bonds with Pro^11^ of tropocollagen, as depicted in Figure S1B. The minimum energy conformation suggested a stable and tight binding between tropocollagen and C4-S.


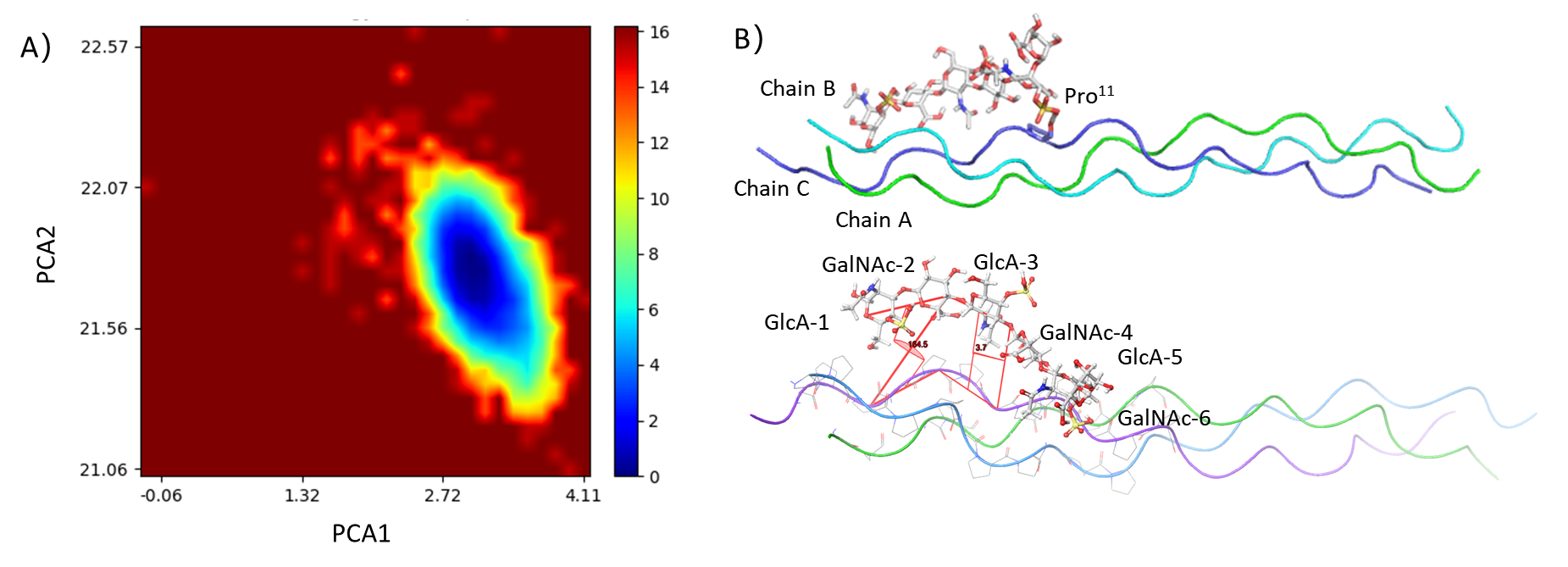


**Figure S1** (A) Free energy landscape of tropocollagen with C4-S complex. (B) Representative minimum energy conformation of tropocollagen + C4-S complex. Chains A, B, and C of tropocollagen are depicted as green, blue, and purple cartoons, while C4-S is shown as gray sticks.


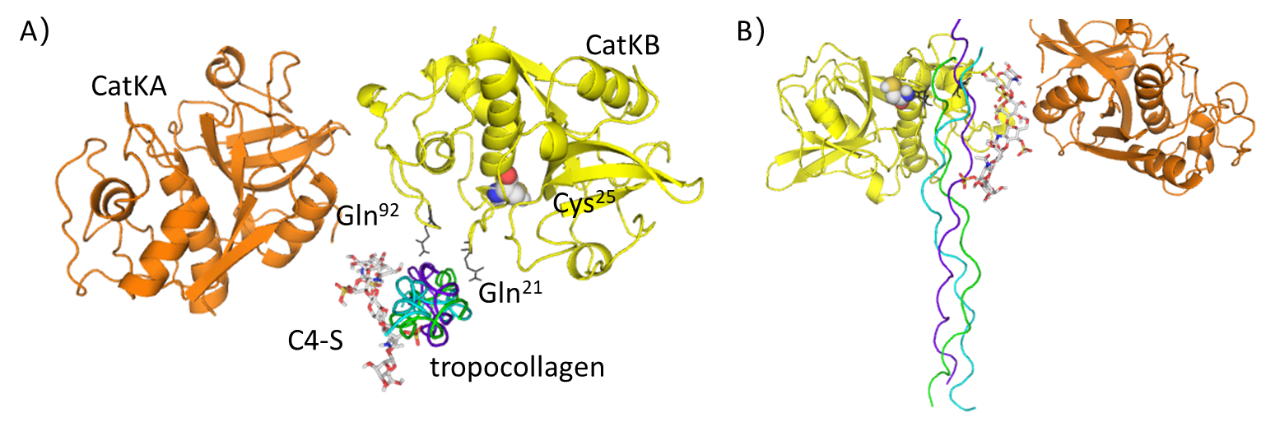


**Figure S2** Structural construction for CatK dimer + tropocollagen + C4-S model. The model in A is rotated 180° around the vertical axis and 180° around the horizontal axis to B. CatKA and CatKB are shown as orange and yellow cartoons, respectively. Chains A, B, and C of tropocollagen are depicted as green, blue, and purple cartoons, while C4-S is shown as gray sticks. Residues Gln^21^ and Gln^92^ are shown in dark grey lines, and sphere denotes the Cys^25^.

In the dynamics process, the hydroxyl and sulfuric acid groups in C4-S formed water bridges with a large number of Pro and Gly residues in tropocollagen (Figure S3A). Furthermore, the hydroxyl and sulfate groups were capable of forming hydrogen bonds with specific residues such as Gly^24^ of ChainB and Gly^12^ of ChainC. Hydrophobic groups like methyl groups in C4-S structure could engage in hydrophobic interactions with the hydrophobic regions of tropocollagen, exemplified by residues Pro^5^ of ChainB and Pro^22^ of ChainB. To further elucidate the interactions, the number of hydrogen bonds, hydrophobic interactions, and water bridges between the tropocollagen and C4-S were calculated. As shown in Figure S3B, C4-S and tropocollagen could form many water bridges, a small amount of hydrogen bonds and hydrophobic interactions, with no ionic interactions observed.


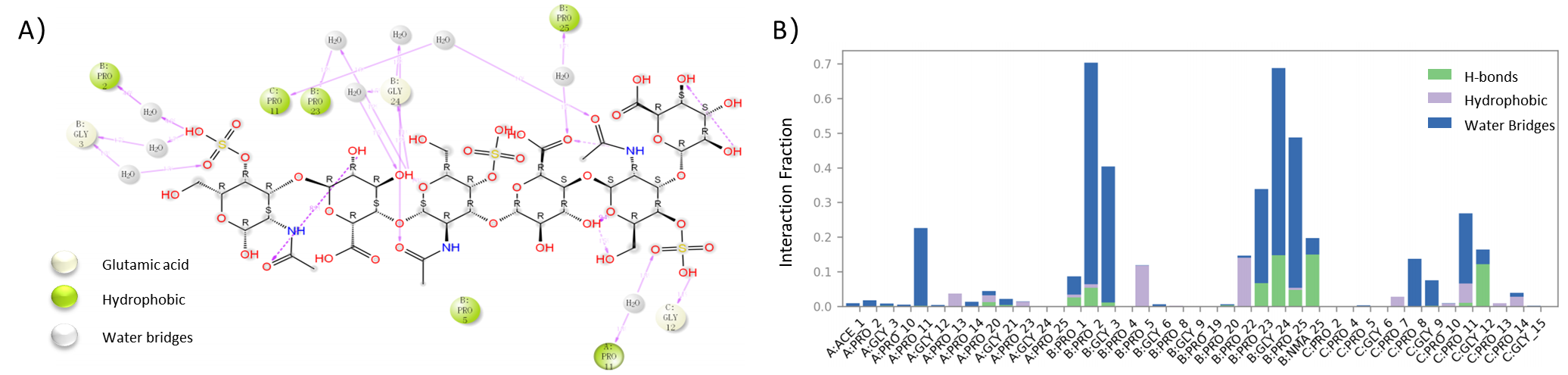


**Figure S3** Molecular dynamics analysis of tropocollagen interactions with the C4-S. (A) 2D interaction diagram from the MD trajectory. The interaction with a cutoff of 10 %. (B) The contacts between tropocollagen and C4-S in MD simulations.

**
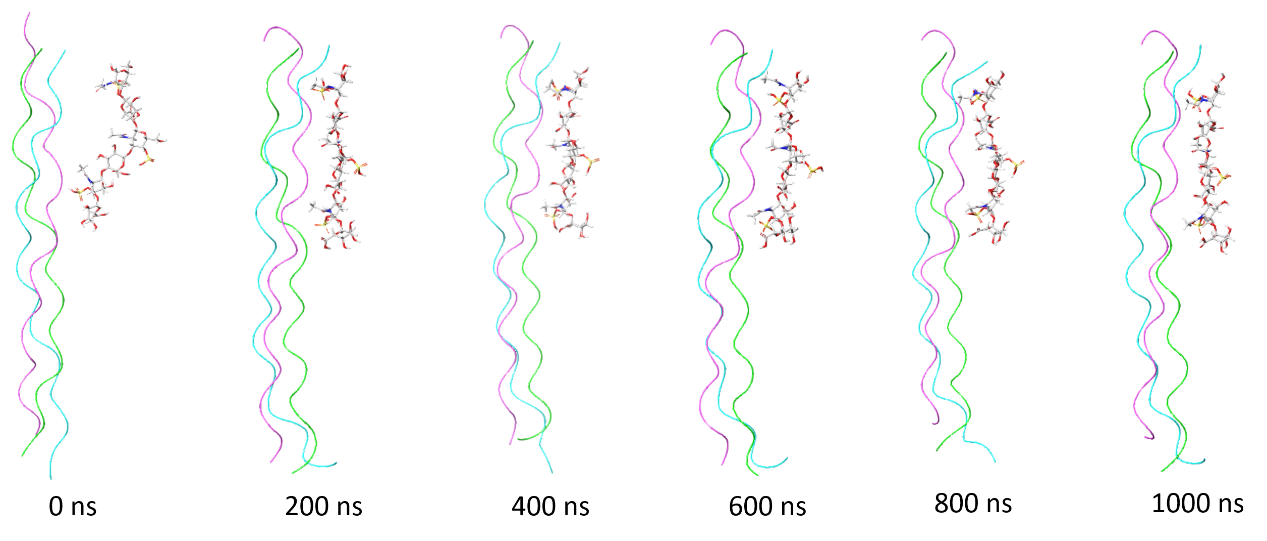
**

**Figure S4** Time-resolved structural evolution of the tropocollagen + C4-S complex during MD simulation (snapshots every 200 ns).

**
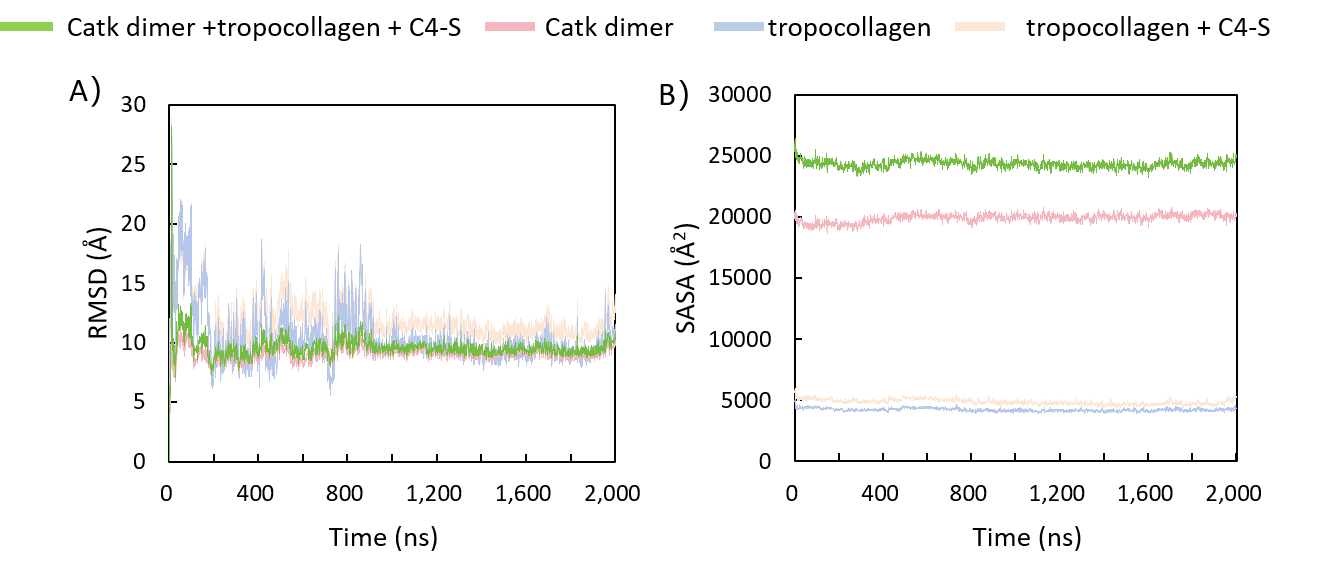
**

**Figure S5** Temporal evolution of RMSD (A) and SASA (B) for CatK dimer + tropocollagen + C4-S ternary complex during a replicate MD simulation. The trajectory values for the CatK dimer + tropocollagen + C4-S complex are represented by a green line, while those for the CatK dimer, tropocollagen, and tropocollagen + C4-S components are shown as pink, blue, and light orange lines, respectively.

**
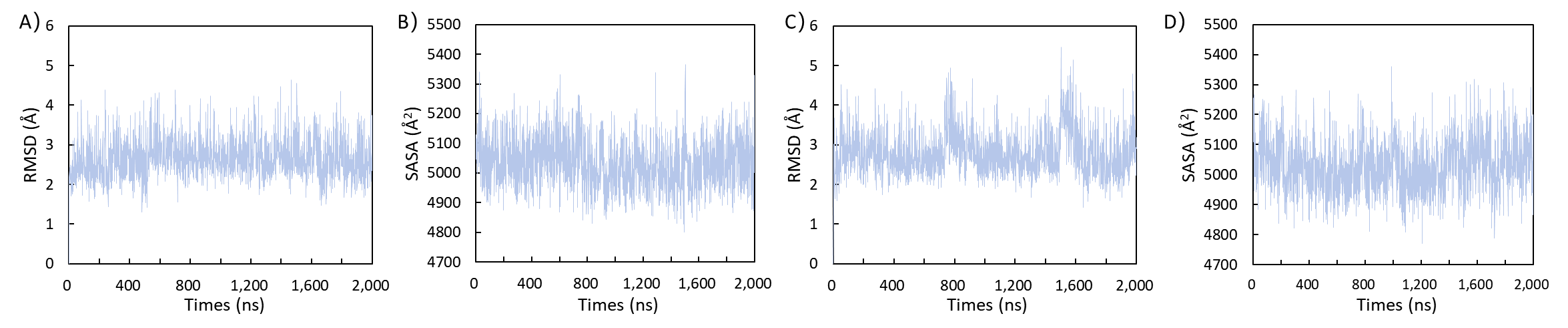
**

**Figure S6** Temporal evolution of RMSD and SASA for tropocollagen system during MD simulations. (A and B) First simulation (run 1), (C and D) Replicate simulation (run 2).

**
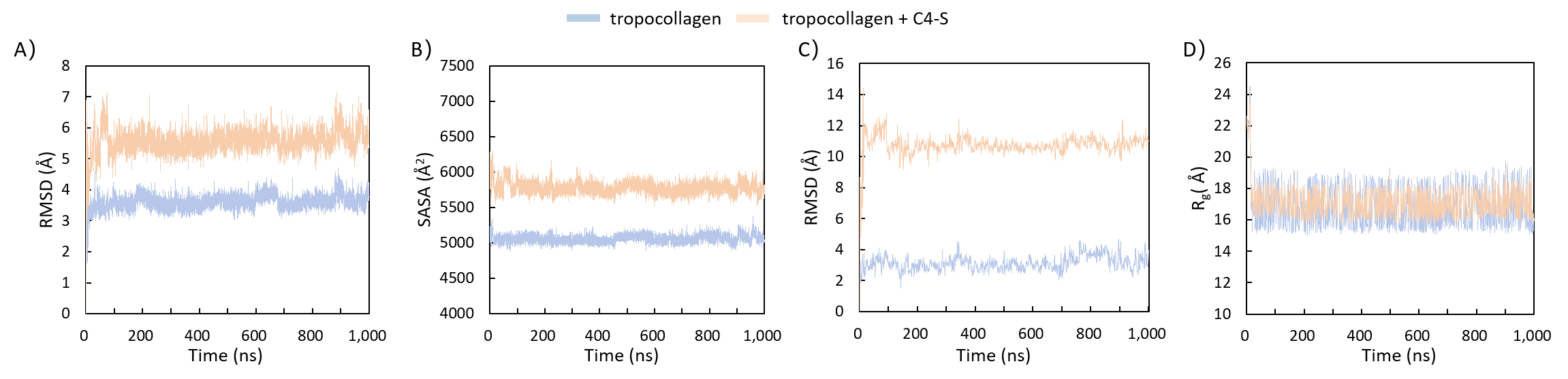
**

**Fig. S7** Temporal evolution of RMSD and SASA for tropocollagen + C4-S binary complex during MD simulations. (A and B) First simulation (run 1), (C and D) Replicate simulation (run 2). The trajectory values for the tropocollagen + C4-S complex are shown as a light orange line, and those for tropocollagen as a blue line.

**
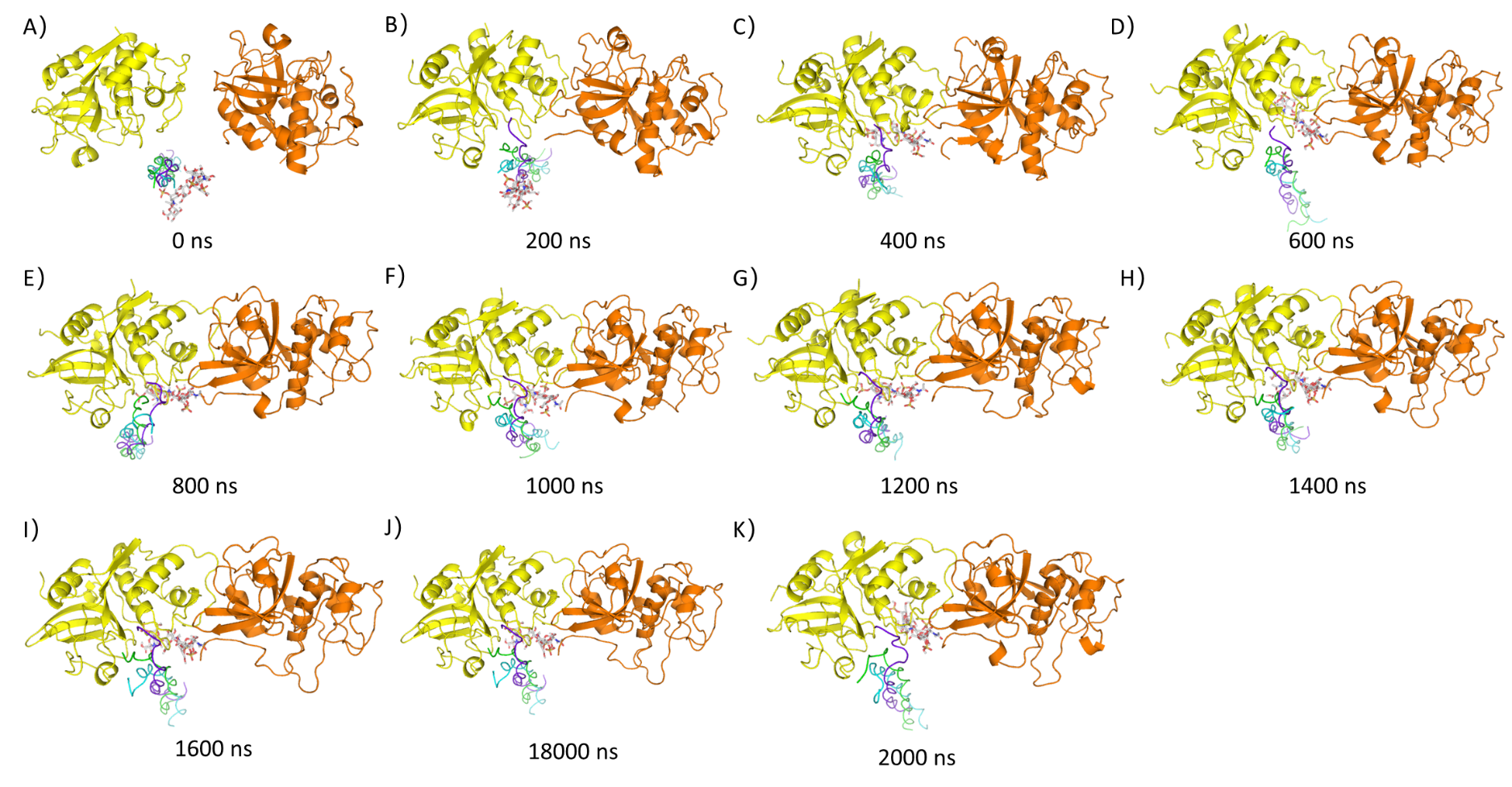
**

**Figure S8** Time-resolved structural evolution of the CatK dimer + tropocollagen + C4-S complex during a replicate MD simulation (snapshots every 200 ns).

The distributions of interatomic distances were compared across MD simulations to investigate the detailed variability of the active site cleft. According to Slovenia *et al.* ^2^ study, the distances and radial distribution Gly^65^ (Cα atom)-Asn^161^ (Cβ atom) and Gly^66^ (O atom)-Leu^160^ (O atom), which were located at the narrowest part of the active site cleft, were selected to characterize the state of the CatK (Figure S9). Given Cys^25^'s pivotal role in disrupting hydrogen bonds between Gly^6^ and Pro^3^, the distance between Gly^6^ (N atom) and Cys^25^ (S atom) was measured. It could also be used to evaluate the distance between the cleft and the Chain C.

The initial nanoseconds of the simulation yielded interatomic distances that were potentially skewed by solvent imbalances, so the values were disregard. Subsequent analysis within the 0-300 ns revealed an expansion of the active site cleft in CatKB, characterized by an increase in the distances between Gly^65^ (Cα atom)-Asn^161^ (Cβ atom) and Gly^66^ (O atom)-Leu^160^ (O atom). The expansion aligned with the significant fluctuations in RMSD and Rg observed during the same period, suggesting that CatK structure manifest a significant propensity for accommodating tropocollagen binding. As a consequence, widening of the groove, with distances expanding to approximately 10.0 Å for Gly^65^ (Cα atom)-Asn^161^ (Cβ atom) and 7.6 Å for Gly^66^ (O atom)-Leu^160^ (O atom), set the stage for efficient substrate degradation. Notably, the interdomain distance between tropocollagen and CatKB contracted from an initial 18.8 Å, suggesting the trends of the tropocollagen domain towards the CatKB groove. After balancing, the distance of Gly^6^ (N atom) and Cys^25^ (S atom) maintained stability, with interactions between Chain C and cleft predominantly occurring within a 10 Å range. It indicated that 10 Å was of a suitable distance for tropocollagen unwinding. A minor rise occurred after 1800 ns, potentially indicating the necessitate additional space for the Chain C to accommodate the structural change with the disruption of a hydrogen bond for Gly^6^. The findings showed that the presence of tropocollagen could affect the stability of the CatK substrate binding site, causing it to expand, and that a distance of around 10 Å between tropocollagen and the CatK substrate binding site was appropriate.


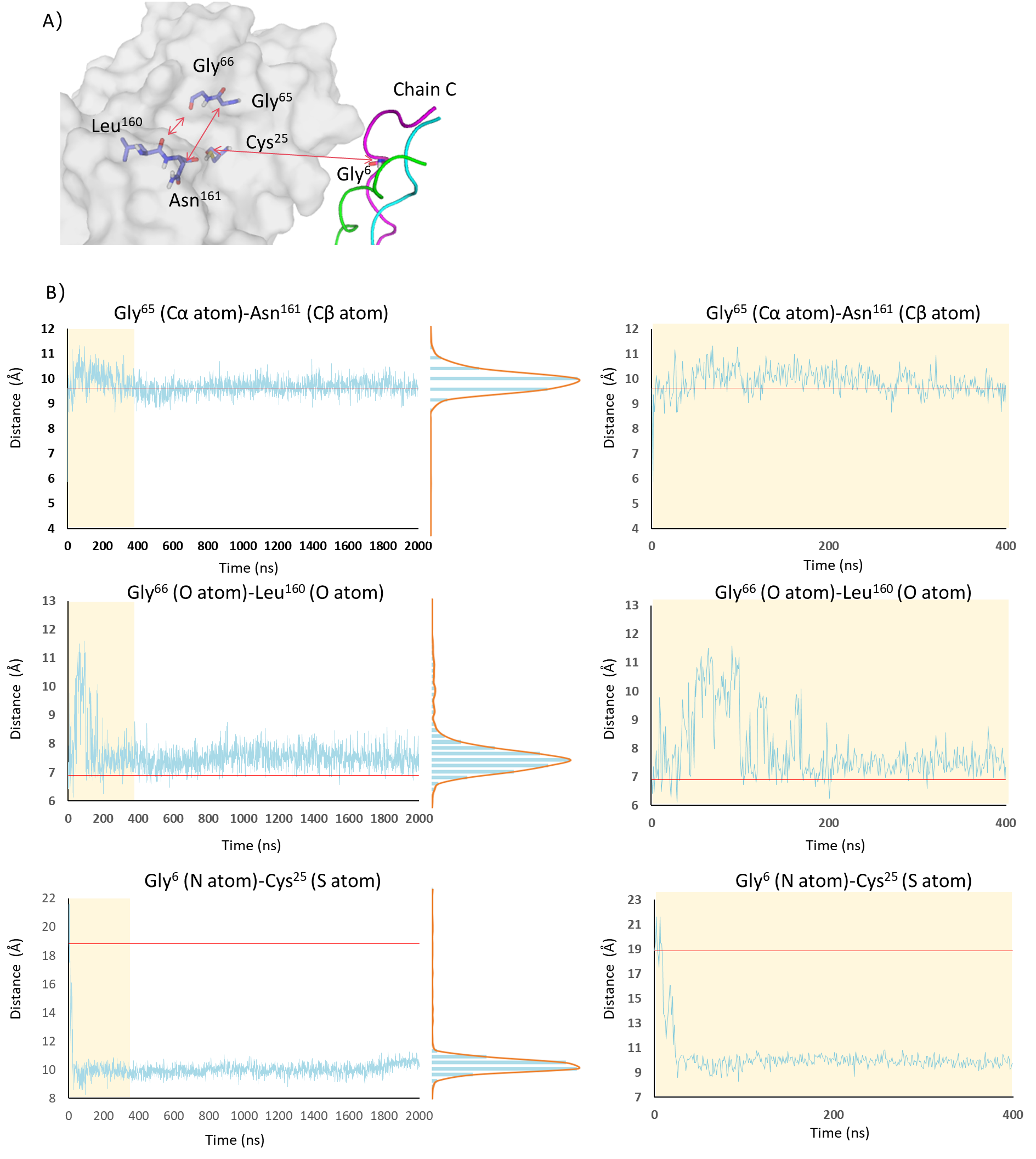


**Figure S9** Tropocollagen binding site geometry and dynamics in CatK. (A) Critical residues (purple sticks) in active site region and tropocollagen binding site of CatK. (B) Interatomic distances (left) and radial distribution (middle) for 2 μs, and distances for 0 ns to 400 ns (right) of CatK with tropocollagen in MD simulations. Red line presents the original distance in the model.


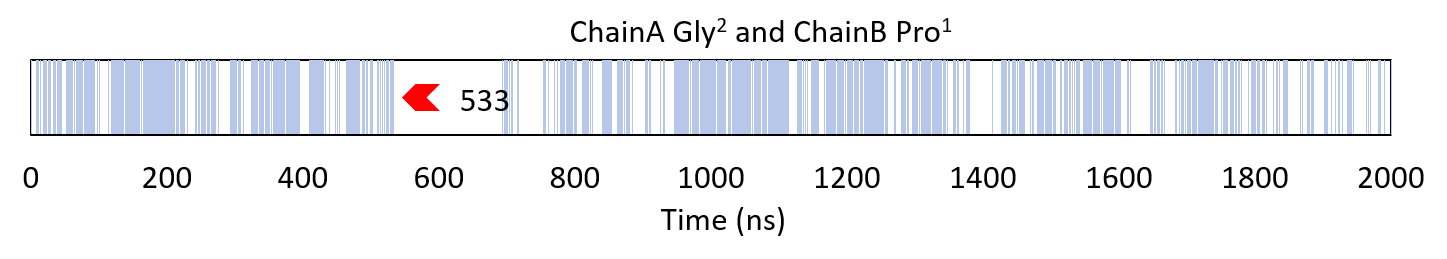


**Figure S10** Temporal evolution and persistent disruption of interchain hydrogen bonds within the tropocollagen triple helix during the 2 μs MD simulation of the tropocollagen system. Red arrows mark the onset of persistent disruption (defined as a break lasting ≥100 ns), with the specific time (ns) labeled.


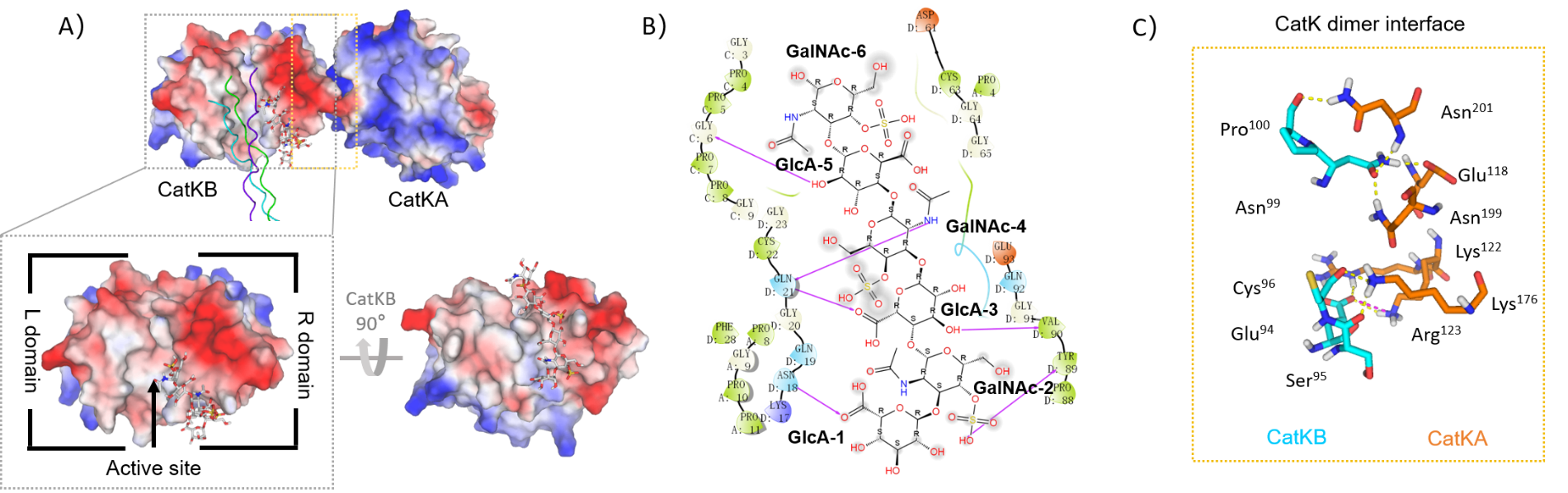


**Figure S11** (A) Electrostatic surface potential of the CatK dimer (structure taken from CatK dimer + tropocollagen + C4-S complex in last frame, but calculated without tropocollagen or C4-S). The visualization of C4-S on the CatKB electrostatic surface was enlarged and rotated by 90°. The CatK is shown as a surface representation, tropocollagen as a cartoon, and C4-S as gray sticks. Negative potential regions are in red, positive potential regions in blue. (B) 2D interaction diagram of C4-S within CatK dimer + tropocollagen + C4-S at the final 1 nanosecond of the dynamics process. (C) The contacts between CatKA (right) and CatKB (left). Blue sticks represent CatKB interacting residues, orange sticks represent CatKA interacting residues, yellow dashed lines indicate hydrogen bonds, and purple dashed lines indicate salt bridges.


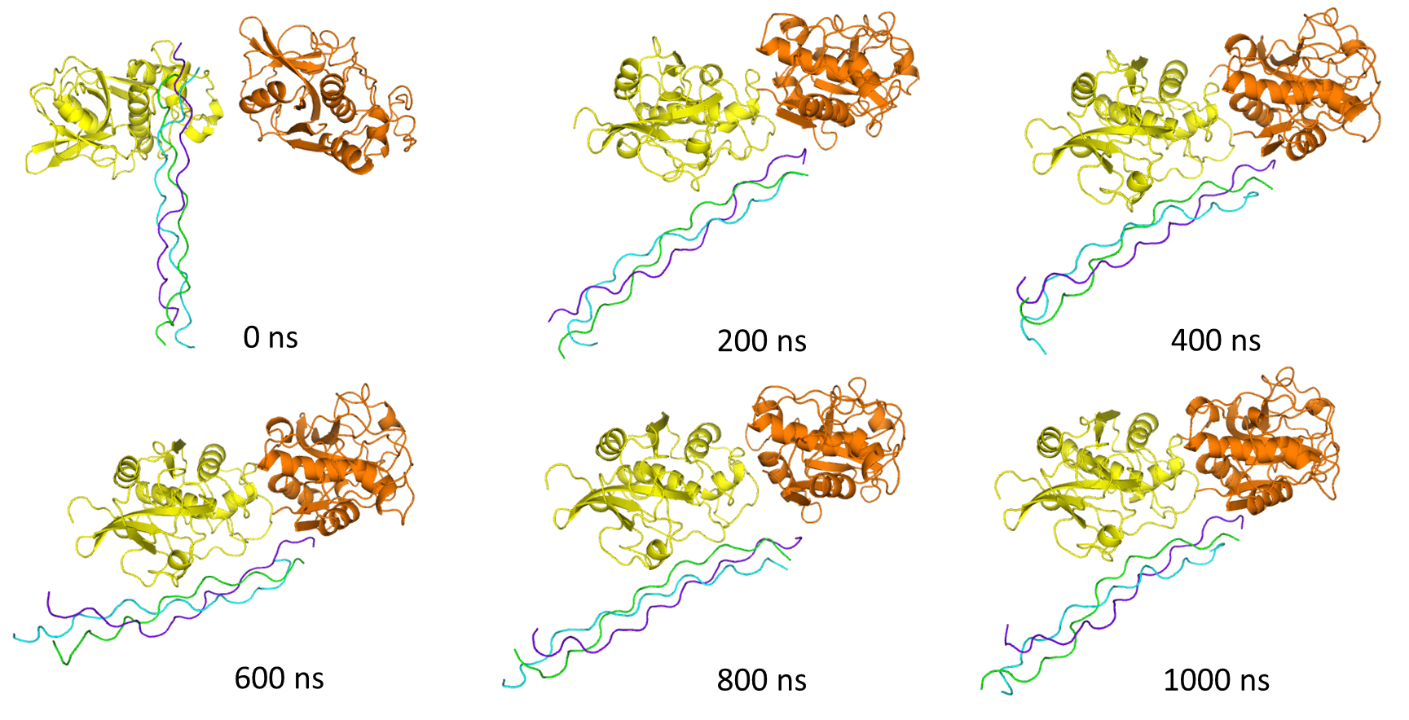


**Figure S12** Time-resolved structural evolution of the CatK dimer + tropocollagen complex during MD simulation (snapshots every 200 ns). The model was based on the CatK dimer + tropocollagen + C4-S model with C4-S removed, other parameters remained consistent. The orientation of snapshots was the same as in Figure S2B.

To investigate the impact of C4-S absence on the complex's structural and dynamics of the assembly, we have modeled a CatK dimer + tropocollagen complex without C4-S and performed a 1 μs MD simulation (Figure S13). In the absence of C4-S, the RMSF values of the CatK dimer remained below 8.0 Å. Apart from internal bonds in the tropocollagen, Chain C interacted mainly with these segments of the CatK dimer: Asn^18^-Cys^22^ and Val^90^-Glu^93^ on CatKB, and Tyr^110^-Asn^117^ on CatKA (green column in Figure S13A). As illustrated in Figure S13B, interactions with the CatK dimer featured many hydrogen bonds and water bridges at Gln^21^ and Gln^92^ of CatKB, as well as Arg^111^ and Glu^112^ of CatKA, along with limited hydrophobic interactions at Tyr^110^ and Pro^114^ of CatKA. Figure S13C showed that, after Pro^5^, the hydrogen bonds formed between proline and glycine maintained over 60% occupancy, implying a reduced tropocollagen unwinding rate in the absence of C4-S. In general, lacking C4-S led to CatK dimer instability, weakened CatKB restraint on tropocollagen, enhanced CatKA with tropocollagen contacts, and decelerated unwinding.


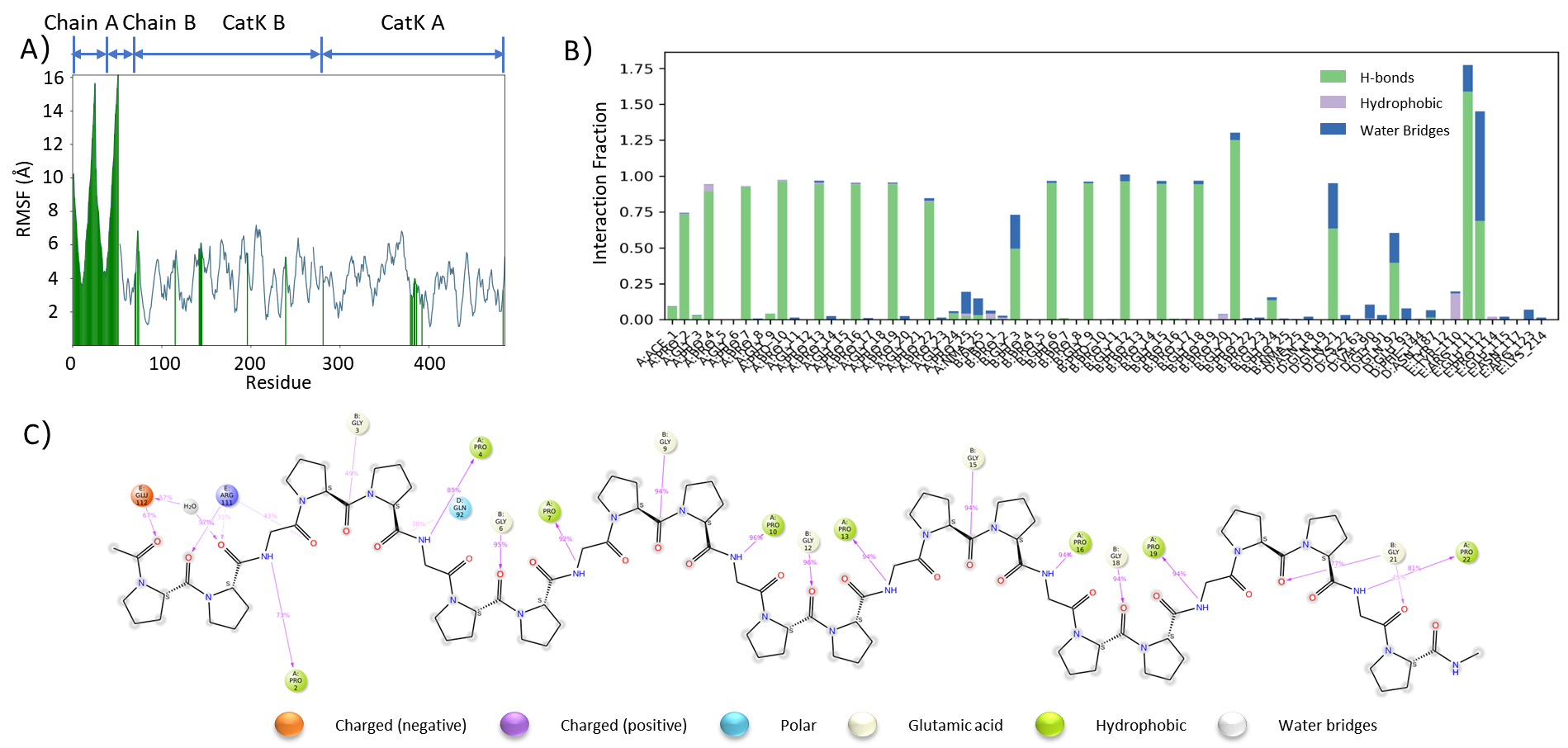


**Figure S13** Molecular dynamics analysis of tropocollagen Chains A, B and CatK dimer interactions with Chain C in CatK dimer + tropocollagen model. (A) RMSF in MD simulations of Chains A, B and CatK dimer, which Chain C as ligand. Protein residues that interact with the ligand are marked with green column. (B) Protein−ligand contacts during MD simulations. (C) 2D interaction diagram from the MD trajectory. The interaction with a cutoff of 30 %.

The RMSF values were obtained by comparing the transient positions of residues to their average positions. Figure S14 illustrated that the CatK dimer showed RMSF values below 5 Å. Within the CatK dimer, Chain C primarily interacted with the following regions (green column in Figure S14A): Gln^19^-Cys^25^, Asp^61^-Thr^69^, Ser^157^-Ala^163^ and Ser^183^-Asn^187^ of CatKB (predominantly involving the active site), as well as Asp^61^-Gly^64^ and Ala^86^-Thr^101^ of CatKA. Specifically, interactions with CatKB involved numerous water bridges with Gly^64^, Tyr^67^, Asn^159^, Leu^160^, and Asn^161^, hydrogen bonds with Gly^66^, Leu^160^, and Asn^161^, and minor hydrophobic contacts with Tyr^67^ and Trp^184^ (Figure S14B). In contrast, interactions with CatKA were dominated by hydrophobic contacts with Tyr^87^, Pro^88^, Val^90^, Met^97^, and Tyr^98^, supplemented by a limited number of hydrogen bonds with Gln^92^, Ser^95^, and Tyr ^98^.


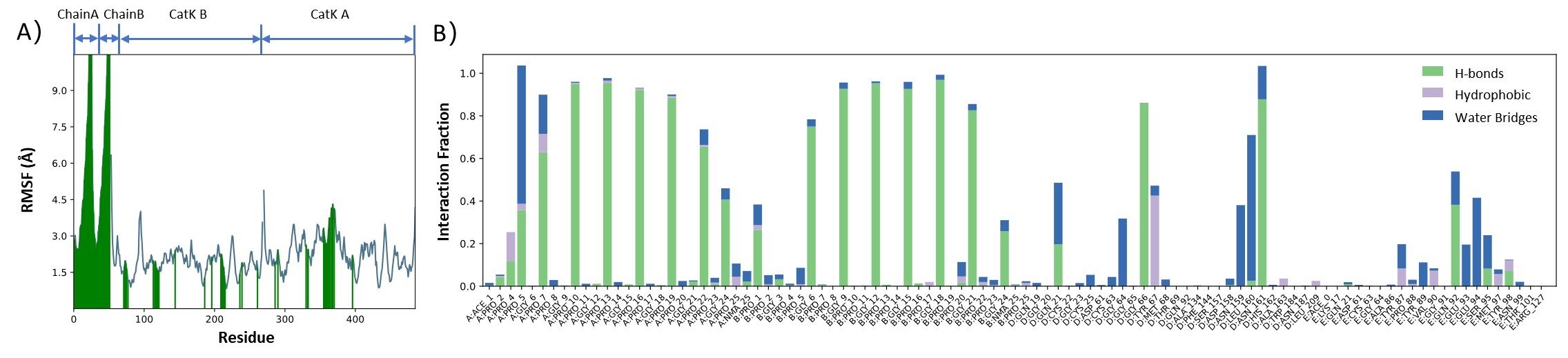


**Figure S14** Molecular dynamics analysis of tropocollagen Chains A, B and CatK dimer interactions with Chain C in CatK dimer + tropocollagen + C4-S model. (A) RMSF in MD simulations of Chains A, B and CatK dimer, which Chain C as ligand. Protein residues that interact with the ligand are marked with green column. (B) Protein−ligand contacts during MD simulations.

**Table S1** The ΔΔ*G* values for alanine mutants of key functional CatK residues.

| Mutations | ΔΔ*G* | Coulomb | Hbond | Lipo | Solv GB | vdW |
| --- | --- | --- | --- | --- | --- | --- |
| 19 (Gln>Ala) | 1.54 | 0.75 | 0 | 0.63 | -1.05 | 1.22 |
| 23 (Gly>Ala) | 1.55 | -2.02 | 0 | -1.67 | 0.6 | 4.65 |
| 25 (Cys>Ala) | 1.08 | -0.03 | 0 | -0.1 | 0.02 | 1.19 |
| 61 (Trp>Ala) | 0.5 | 0.3 | 0 | 0.32 | -1.24 | 1.11 |
| 67 (Tyr>Ala) | 7.09 | 0.91 | 0 | 4.59 | -1.59 | 3.17 |
| 162 (Hie>Ala) | 1.95 | 0.29 | 0 | 0.49 | 0.14 | 1.03 |
| 184 (Trp>Ala) | 12.22 | 0.17 | 0 | 6.08 | -1.13 | 7.1 |
| 209 (Leu>Ala) | 3.22 | -0.04 | 0 | 1.99 | 0 | 1.27 |

ΔΔ *G* (kcal/mol) is decomposed into contributions from coulombic energy, hydrogen bond (Hbond) energy, hydrophobic interaction (Lipo) energy, generalized Born solvation (Solv GB) energy, and van der Waals (vdW) energy.


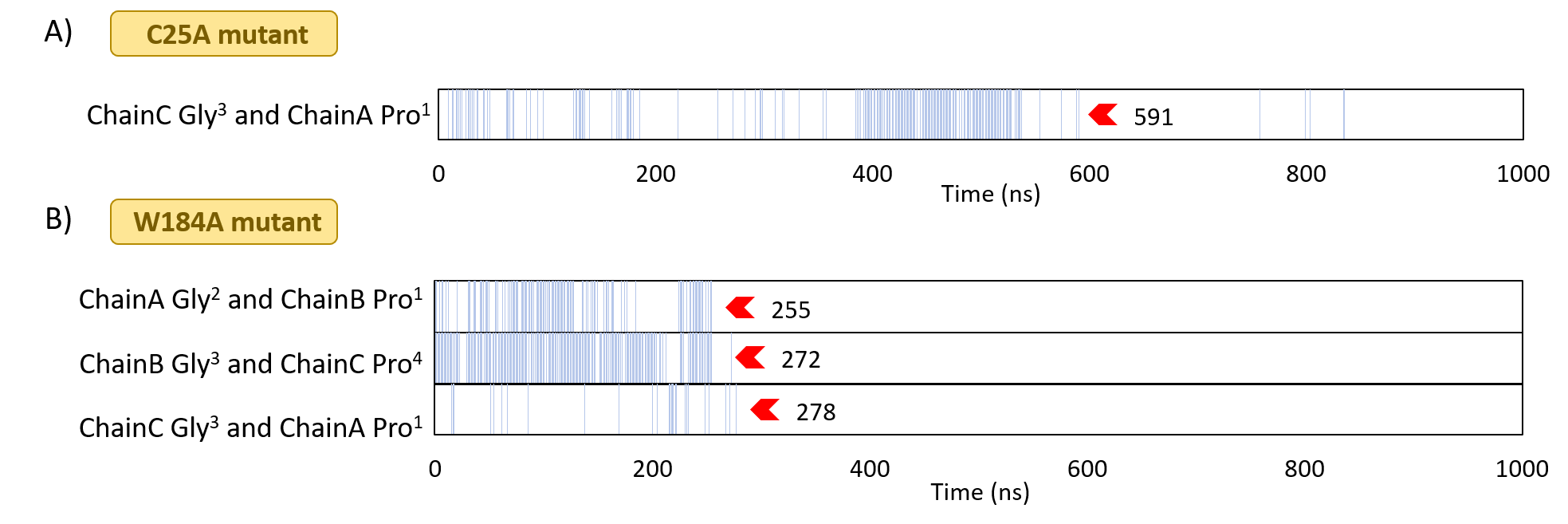


**Figure S15** Temporal evolution and persistent disruption of interchain hydrogen bonds within the tropocollagen triple helix during the 2 μs MD simulation of the CatKA + mutant CatKB + tropocollagen + C4-S system. Red arrows mark the onset of persistent disruption (defined as a break lasting ≥100 ns), with the specific time (ns) labeled.

**Table S2** The protein-protein contacts between CatKA and CatKB within CatK dimer + tropocollagen + C4-S system.

| Interaction | CatKB | CatKA | Distance (Å) | Number |
| --- | --- | --- | --- | --- |
| H-bond | Pro^100^ | Asn^201^ | 2.1 | 1 |
| H-bond | Asn^99^ | Asn^201^ | 2.3 | 1 |
| H-bond | Asn^99^ | Glu^118^ | 2.0 | 1 |
| H-bond | Asn^99^ | Asn^199^ | 2.0 | 1 |
| H-bond | Cys^96^ | Lys^176^ | 2.2 | 1 |
| H-bond | Ser^95^ | Lys^176^ | 2.6 | 1 |
| Salt bridge | Glu^94^ | Lys^122^ | 3.3 | 1 |
| H-bond | Glu^94^ | Lys^122^ | 2.4 | 1 |
| H-bond | Glu^94^ | Arg^123^ | 2.3 and 2.4 | 2 |


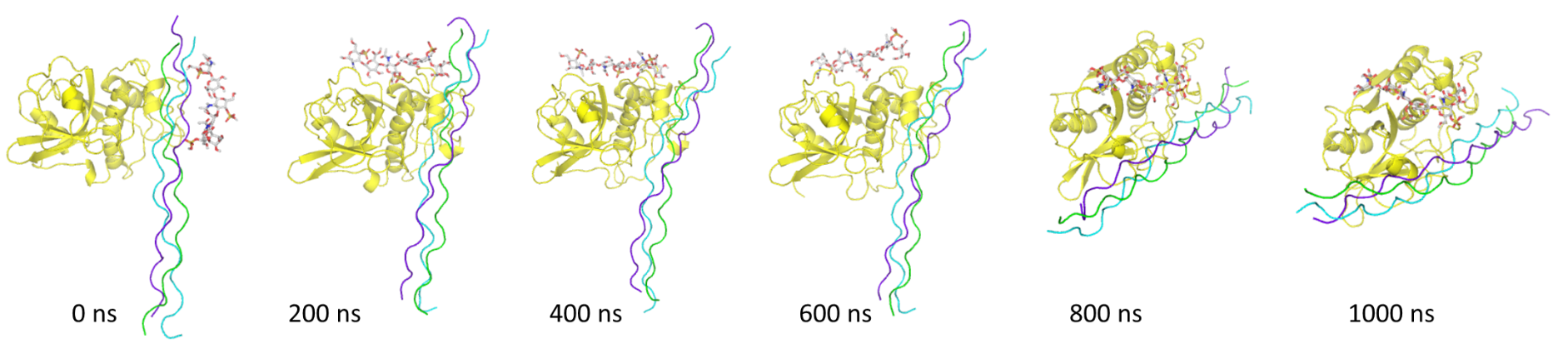


**Figure S16** Time-resolved structural evolution of the CatKB + tropocollagen + C4-S complex during MD simulation (snapshots every 200 ns). The model was based on the CatK dimer + tropocollagen + C4-S model with CatKA removed, other parameters remained consistent. The orientation of snapshots was the same as in Figure S2B.


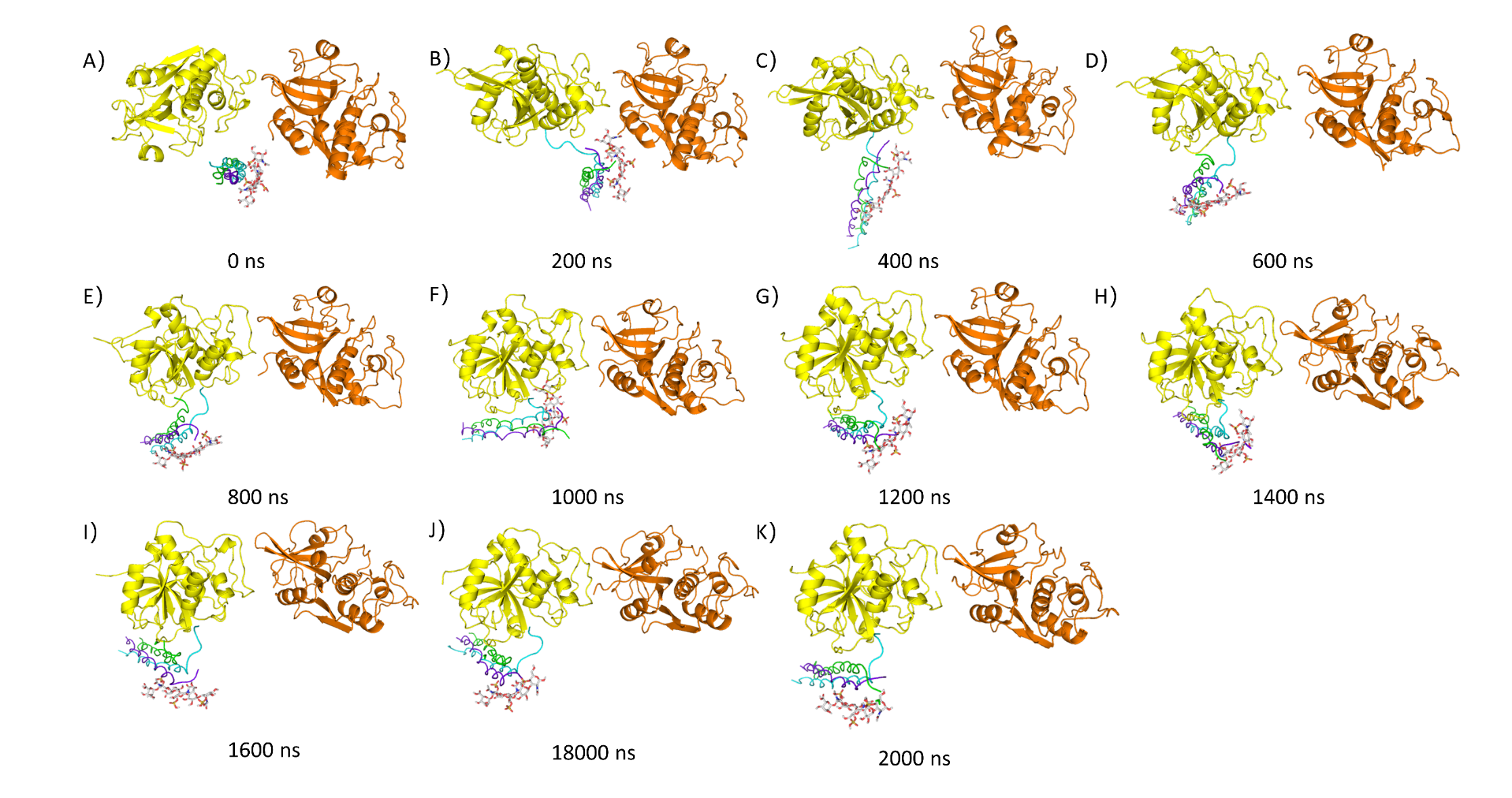


**Figure S17** Structural snapshots of CatK dimer + tropocollagen_Gly-Pro-Hyp_ + C4-S system at 200 ns intervals. CatKA and CatKB are shown in orange and yellow, respectively. The tropocollagen consisted of Gly-Pro-Hyp motifs extracted from PDB ID 7CWK.


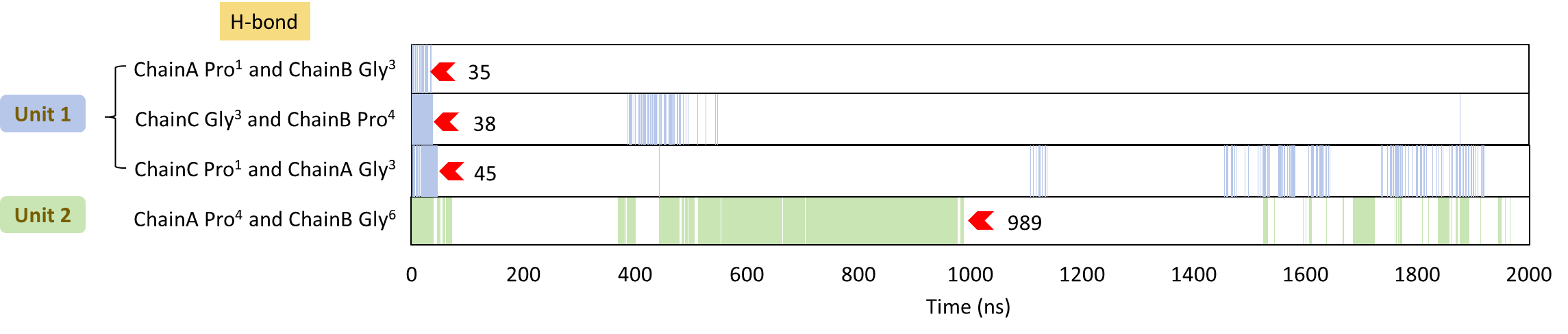


**Figure S18** Temporal evolution and persistent disruption of interchain hydrogen bonds within the tropocollagen triple helix during the 2 μs MD simulation of the CatK dimer + tropocollagen_Gly-Pro-Hyp_ + C4-S system. Red arrows mark the onset of persistent disruption (defined as a break lasting ≥100 ns), with the specific time (ns) labeled.

**Table S3** The protein-protein contacts between CatKA and CatKB within CatK dimer + tropocollagen_Gly-Pro-Hyp_ + C4-S system.

| Interaction | CatKB | CatKA | Distance (Å) | Number |
| --- | --- | --- | --- | --- |
| H-bond | Ser^95^ | Asp^3^ | 2.0 | 1 |
| H-bond | Glu^94^ | Lys^176^ | 2.0 | 1 |
| H-bond | Glu^94^ | Lys^122^ | 1.9 | 1 |
| Salt bridge | Glu^94^ | Lys^122^ | 2.3 | 1 |
| H-bond | Glu^94^ | Arg^198^ | 2.3 and 2.4 | 2 |
| Salt bridge | Glu^94^ | Arg^198^ | 2.9 | 1 |
| H-bond | Glu^92^ | Lys^119^ | 2.2 | 1 |

**References**

(1) Tiwary, P.; Parrinello, M. A Time-Independent Free Energy Estimator for Metadynamics. *J. Phys. Chem. B* **2015**, *119* (3), 736-742.

(2) Novinec, M. Computational investigation of conformational variability and allostery in cathepsin K and other related peptidases. *PLoS One* **2017**, *12* (8), e0182387.

(3) Aguda, A. H.; Panwar, P.; Du, X.; Nguyen, N. T.; Brayer, G. D.; Brömme, D. Structural basis of collagen fiber degradation by cathepsin K. *P. Natl. Acad. Sci. U. S. A.* **2014**, *111* (49), 17474-17479.
